# Prion protein protects mice from lethal infection with influenza A viruses

**DOI:** 10.1371/journal.ppat.1007049

**Published:** 2018-05-03

**Authors:** Junji Chida, Hideyuki Hara, Masashi Yano, Keiji Uchiyama, Nandita Rani Das, Etsuhisa Takahashi, Hironori Miyata, Yukiko Tomioka, Toshihiro Ito, Hiroshi Kido, Suehiro Sakaguchi

**Affiliations:** 1 Division of Molecular Neurobiology, Institute for Enzyme Research (KOSOKEN), Tokushima University, Tokushima, Japan; 2 Division of Enzyme Chemistry, Institute for Enzyme Research, Tokushima University, Tokushima, Japan; 3 Animal Research Center, School of Medicine, University of Occupational and Environmental Health, Kitakyushu, Japan; 4 Avian Zoonosis Research Center, Faculty of Agriculture, Tottori University, Koyama-cho, Tottori, Japan; University of Georgia, UNITED STATES

## Abstract

The cellular prion protein, designated PrP^C^, is a membrane glycoprotein expressed abundantly in brains and to a lesser extent in other tissues. Conformational conversion of PrP^C^ into the amyloidogenic isoform is a key pathogenic event in prion diseases. However, the physiological functions of PrP^C^ remain largely unknown, particularly in non-neuronal tissues. Here, we show that PrP^C^ is expressed in lung epithelial cells, including alveolar type 1 and 2 cells and bronchiolar Clara cells. Compared with wild-type (WT) mice, PrP^C^-null mice (*Prnp*^*0/0*^) were highly susceptible to influenza A viruses (IAVs), with higher mortality. Infected *Prnp*^*0/0*^ lungs were severely injured, with higher inflammation and higher apoptosis of epithelial cells, and contained higher reactive oxygen species (ROS) than control WT lungs. Treatment with a ROS scavenger or an inhibitor of xanthine oxidase (XO), a major ROS-generating enzyme in IAV-infected lungs, rescued *Prnp*^*0/0*^ mice from the lethal infection with IAV. Moreover, *Prnp*^*0/0*^ mice transgenic for PrP with a deletion of the Cu-binding octapeptide repeat (OR) region, Tg(PrPΔOR)/*Prnp*^*0/0*^ mice, were also highly susceptible to IAV infection. These results indicate that PrP^C^ has a protective role against lethal infection with IAVs through the Cu-binding OR region by reducing ROS in infected lungs. Cu content and the activity of anti-oxidant enzyme Cu/Zn-dependent superoxide dismutase, SOD1, were lower in *Prnp*^*0/0*^ and Tg(PrPΔOR)/*Prnp*^*0/0*^ lungs than in WT lungs. It is thus conceivable that PrP^C^ functions to maintain Cu content and regulate SOD1 through the OR region in lungs, thereby reducing ROS in IAV-infected lungs and eventually protecting them from lethal infection with IAVs. Our current results highlight the role of PrP^C^ in protection against IAV infection, and suggest that PrP^C^ might be a novel target molecule for anti-influenza therapeutics.

## Author summary

Influenza A virus (IAV) is an enveloped, negative sense, single-stranded RNA virus, causing seasonal epidemic outbreaks of influenza. Anti-influenza agents targeting viral molecules, such as neuraminidase inhibitors, are currently available. However, these agents have accelerated emergence of mutant IAVs that are resistant to these agents among human populations. Development of new types of anti-influenza agents is awaited. We show that the cellular prion protein PrP^C^ has a protective role against lethal infection with IAVs through the octapeptide repeat (OR) region by abrogating lung epithelial cell apoptosis induced by reactive oxygen species (ROS) in infected lungs. We also show that PrP^C^ could reduce ROS in IAV-infected lungs through the OR region by maintaining Cu ion homeostasis and thereby activating Cu/Zn-dependent superoxide dismutase, SOD1. These results highlight the protective role of PrP^C^ in IAV infection. Elucidation of the exact mechanism underlying the PrP^C^-mediated protection against IAV infection would be important for further understanding the pathogenesis of IAV infection and could be useful for development of new types of anti-influenza therapeutics.

## Introduction

The normal cellular prion protein, designated PrP^C^, is a membrane glycoprotein tethered to the outer cell membrane via a glycosylphosphatidylinositol anchor moiety and expressed most abundantly in brains, particularly by neurons, and to a lesser extent in non-neuronal tissues including hearts, kidneys, and lungs [1,2]. Conformational conversion of PrP^C^ into the abnormally folded, amyloidogenic isoform is a pivotal pathogenic event in prion diseases, a group of neurodegenerative disorders including Creutzfeldt-Jakob disease in humans and scrapie and bovine spongiform encephalopathy in animals [2]. In brains, glial cells including microglia, astrocytes, and oligodendrocytes also express PrP^C^ [3–7]. PrP^C^ expression has also been reported in non-cardiomyocytes in hearts [8], in glomeruli, proximal convoluted tubules and collecting ducts in kidneys [8,9], activated hepatic stellate cells in livers [10], in lymphoid nodules [11] and perilymphoid zones of the red pulp [8] in spleens, and in neuronal cells in the lamina propia and parasympathetic ganglions [8], some epithelial cells [12], Peyer’s patches [12] and enteric glial cells [13] in intestines. In lungs, alveolar walls were reported to be positive for PrP^C^ expression [8]. However, the exact function of PrP^C^ remains to be clarified.

Neuroprotective function has been suggested for PrP^C^. Mice devoid of PrP^C^ (*Prnp*^*0/0*^) have been reported to be vulnerable to ischemic brain injury, with enhanced neuronal cell apoptosis in the injured brains [14–16]. PrP lacking the octapeptide repeat (OR) region failed to rescue *Prnp*^*0/0*^ mice from ischemic brain injury [17]. These results suggest that PrP^C^ might exert an anti-apoptotic activity through the OR region, thereby protecting neurons from ischemic damage. It was recently reported that the hearts and kidneys of *Prnp*^*0/0*^ mice were also vulnerable to ischemic injury [18,19], indicating that PrP^C^ could have a protective function even in non-neuronal tissues. However, the exact mechanism underlying the protective function of PrP^C^ remains elusive.

The OR region binds to Cu ions via histidine residues [20–22]. Some investigators showed that Cu content was reduced and the enzymatic activity of anti-oxidant enzyme Cu/Zn-dependent superoxide dismutase, SOD1, was lower in the brains of *Prnp*^*0/0*^ mice [20,23,24], suggesting that PrP^C^ might function to maintain Cu levels, thereby regulating SOD1 activity to exert anti-oxidative activity and eventually protecting neurons from apoptosis. However, others reported normal levels of Cu content and SOD1 activity in the brains of *Prnp*^*0/0*^ mice [25]. Thus, the role of PrP^C^ in maintenance of Cu content and regulation of SOD1 in terms of its protective activity remains to be determined.

Several groups have investigated the role of PrP^C^ in virus infection in mice [26–28]. Nasu-Nishimura et al. reported that PrP^C^ could have a protective role against infection with encephalomyocarditis virus B variant by reducing neuronal apoptosis in the brains of infected mice without affecting viral replication [27]. It was also reported that human immunodeficiency virus type 1 (HIV-1) production was strongly inhibited by expression of PrP^C^ in cultured cells transfected with an infectious HIV-1 molecular clone [28]. On the other hand, PrP^C^ overexpression was shown to enhance acute infection of herpes simplex virus type 1 (SC16) in the central and peripheral neuronal tissues, causing higher mortality in mice, although latent infection of the virus in these tissues was inhibited by overexpression of PrP^C^ [26].

Influenza A virus (IAV) is an enveloped, negative sense, single-stranded RNA virus, causing seasonal epidemic outbreaks of influenza [29]. High morbidity and mortality are observed in infected people, particularly in the young and elderly and those with underlying chronic diseases in lung or cardiovascular systems [29]. Several lines of evidence indicate that reactive oxygen species (ROS) play a pivotal role in IAV infection-induced lung injury, by causing apoptosis in infected lung epithelial cells [30–33]. However, the role of PrP^C^ in IAV infection remains unknown.

In the present study, we show that *Prnp*^*0/0*^ mice were highly susceptible to IAV infection, with higher mortality, compared to wild-type (WT) mice. PrP lacking the Cu-binding OR region failed to rescue *Prnp*^*0/0*^ mice from lethal infection with IAV. Infected *Prnp*^*0/0*^ lungs were severely injured, with higher epithelial cell apoptosis and higher ROS levels than control WT lungs. Treatment with anti-oxidants rescued *Prnp*^*0/0*^ mice from lethal infection with IAV. SOD1 activity and Cu ion content were lower in *Prnp*^*0/0*^ lungs than in WT lungs. These results suggest that PrP^C^ could have a protective role against lethal infection with IAVs through the OR region probably by exerting an anti-oxidative activity by maintaining Cu content and regulating SOD1 in lungs.

## Results

### PrP^C^ is expressed in lung epithelial cells

**Fig 1:**
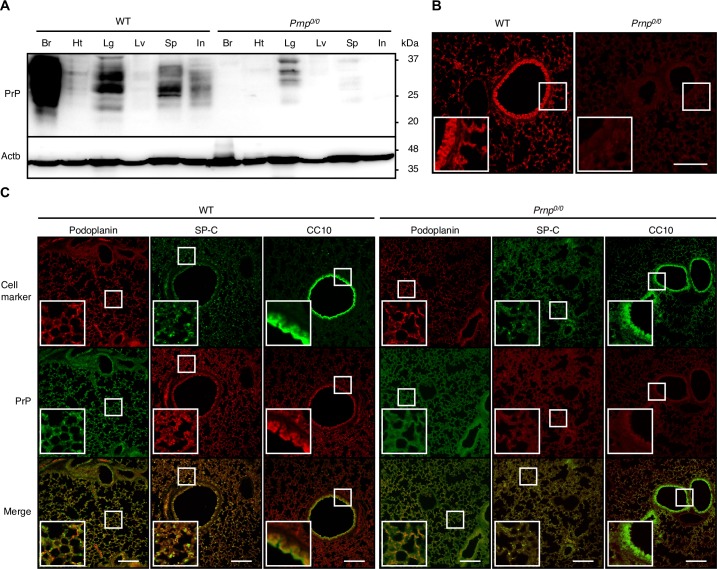
PrP^C^ is expressed in lung epithelial cells. (A) Western blotting of various tissues from C57BL/6 WT and *Prnp*^*0/0*^ mice with 6D11 anti-PrP antibody, which recognizes residues 93–109 of mouse PrP. Non-specific weak signals were observed in lungs and spleens. Actb is an internal control. Br, brain; Ht, heart; Lg, lung; Lv, liver; Sp, spleen; In, intestine. (B) Immunofluorescence staining of WT and *Prnp*^*0/0*^ lungs with IBL-N anti-PrP antibodies, which are raised against a synthetic N-terminal peptide. Bar, 400 μm. Insets show 2 times-magnified images of white squares. (C) Double immunofluorescence staining of WT and *Prnp*^*0/0*^ lungs with IBL-N anti-PrP antibodies and antibodies against podoplanin, SP-C, or CC10. Bar, 400 μm. Insets show 2.5 times-magnified images of white squares.

We first investigated expression of PrP^C^ in lung tissues of C57BL/6 WT mice on Western blotting. PrP^C^ was detectable in various tissues, with highest expression in brains (Fig 1A). Lower but considerably high levels of PrP^C^ were detected in lungs, followed by that in spleens and intestines (Fig 1A). Only very low level of PrP^C^ was detectable in hearts and livers (Fig 1A). These results are consistent with PrP^C^ being expressed most abundantly in brains and, to lesser extents, in other non-neuronal tissues [1,2]. Weak signals were observed in *Prnp*^*0/0*^ lungs (Fig 1A). However, no signals for PrP^C^ were detectable in the brains of *Prnp*^*0/0*^ mice (Fig 1A), clearly indicating that PrP^C^ expression is absent in *Prnp*^*0/0*^ mice. Therefore, the signals observed in *Prnp*^*0/0*^ lungs are not specific for PrP^C^. PrP^C^ is a glycoprotein with two glycosylation sites, therefore di-, mono-, and un-glycosylated forms of PrP^C^ are being expressed and detected as a broad band on Western blotting. We then performed immunofluorescent staining for PrP^C^ in lungs. No specific signals were detected on *Prnp*^*0/0*^ lung slices (Fig 1B). In contrast, bronchiolar and alveolar epithelial cells on WT lung slices showed positive staining (Fig 1B). Double staining with anti-podoplanin, anti-surfactant protein C (SP-C), or anti-Clara cell 10-kDa protein (CC10) antibodies, which specifically detect alveolar type 1 and 2 epithelial cells (AT1 and AT2 cells) and bronchiolar Clara epithelial cells, respectively, revealed expression of PrP^C^ in these lung epithelial cells (Fig 1C).

### *Prnp*^*0/0*^ mice are highly susceptible to IAV infection

**Fig 2:**
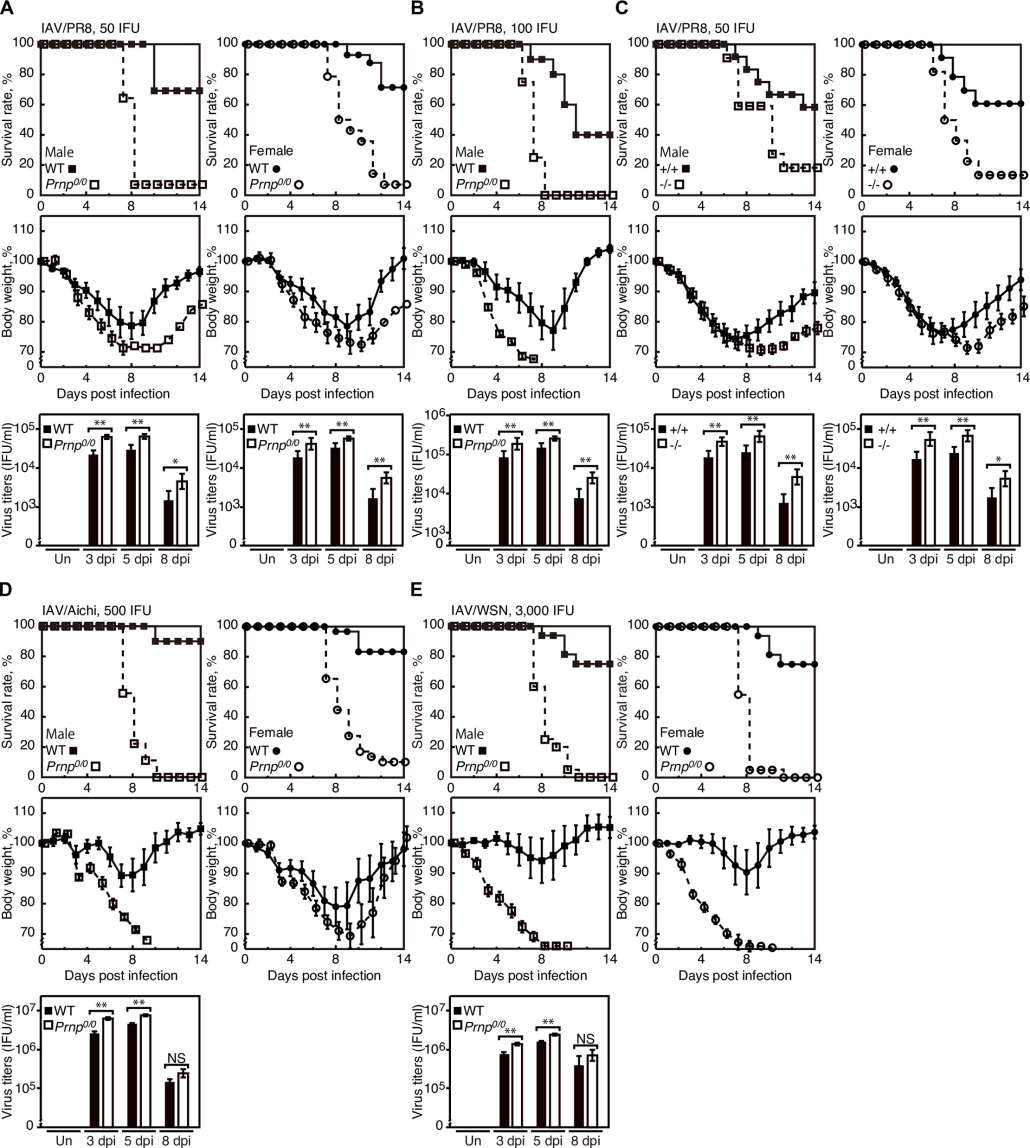
*Prnp*^*0/0*^ mice are highly susceptible to IAV/PR8. (A) Mortality and body weight of male (left panels) and female (right panels) *Prnp*^*0/0*^ and WT mice after intranasal infection with 50 IFU of IAV/PR8 (male *Prnp*^*0/0*^, n = 14; male WT, n = 13; female *Prnp*^*0/0*^, n = 14; female WT, n = 14). The *p* value for mortality: *p*<0.0001 in both genders. Lower panels: Viral titers in the lungs of *Prnp*^*0/0*^ (n = 3) and WT mice (n = 3) uninfected (Un) and infected with IAV/PR8 (50 IFU) at 3, 5, and 8 dpi. (B) Mortality and body weight of male *Prnp*^*0/0*^ (n = 8) and WT (n = 10) mice after intranasal infection with 100 IFU of IAV/PR8. The *p* value for mortality: *p* = 0.0003. Lower panel: Viral titers in the lungs of *Prnp*^*0/0*^ (n = 3) and WT mice (n = 3) uninfected (Un) and infected with IAV/PR8 (100 IFU) at 3, 5, and 8 dpi. (C) Mortality and body weight of male (left panels) and female (right panels) *Prnp*^*0/0*^ and *Prnp*^*+/+*^ littermates after intranasal infection with 50 IFU of IAV/PR8 (male *Prnp*^*0/0*^, n = 22; male *Prnp*^*+/+*^, n = 12; female *Prnp*^*0/0*^, n = 22; female *Prnp*^*+/+*^, n = 23). The *p* value for mortality: *p* = 0.0135 in males, *p*<0.0001 in females. Lower panels: Viral titers in the lungs of *Prnp*^*0/0*^ (n = 3) and *Prnp*^*+/+*^ mice (n = 3) uninfected (Un) and infected with IAV/PR8 (50 IFU) at 3, 5, and dpi. (D) Mortality and body weight of male (left panels) and female (right panels) WT and *Prnp*^*0/0*^ mice after intranasal infection with 500 IFU of IAV/Aichi (male WT, n = 20; male *Prnp*^*0/0*^, n = 9; female WT, n = 30; female *Prnp*^*0/0*^, n = 29). The *p* value for mortality: *p*<0.0001 in both genders. Lower left panel: Viral titers in the lungs of male *Prnp*^*0/0*^ (n = 3) and WT mice (n = 3) uninfected (Un) and infected with IAV/Aichi (500 IFU) at 3, 5, and 8 dpi. (E) Mortality and body weight of male (left panels) and female (right panels) WT and *Prnp*^*0/0*^ mice after intranasal infection with 3,000 IFU of IAV/WSN (male WT, n = 16; male *Prnp*^*0/0*^, n = 20; female WT, n = 16; female *Prnp*^*0/0*^, n = 20). The *p* value for mortality: *p*<0.0001 in both genders. Lower left panel: Viral titers in the lungs of male *Prnp*^*0/0*^ (n = 3) and WT lungs (n = 3) uninfected (Un) and infected with IAV/WSN (3,000 IFU) at 3, 5, and 8 dpi. The 0 dpi in the graphs of survival rate and body weight is 5–10 min after infection. *, p<0.05; **, p<0.01. Error bars, SD.

To investigate the role of PrP^C^ in IAV infection, we intranasally infected *Prnp*^*0/0*^ and C57BL/6 WT mice with 50 infectious units (IFU) of influenza virus strain A/Puerto Rico/8/34 (H1N1) (hereafter referred to as IAV/PR8). Compared to control WT mice, male *Prnp*^*0/0*^ mice showed higher sensitivity to IAV/PR8, with markedly elevated mortality (Fig 2A). At 14 days post-infection (dpi), only about 7% of male *Prnp*^*0/0*^ mice survived the infection while more than 70% of male WT mice were still alive. Higher mortality was also observed in infected female *Prnp*^*0/0*^ mice, compared to control female WT mice (Fig 2A). Viral titers were higher in infected *Prnp*^*0/0*^ lungs than in control WT lungs (Fig 2A). Western blotting showed similar expression of PrP^C^ in male and female WT lungs (S1 Fig). We also intranasally infected male *Prnp*^*0/0*^ and WT mice with increasing infectious doses (100 IFU) of IAV/PR8. None of *Prnp*^*0/0*^ mice survived the infection by 14 dpi (Fig 2B). However, about 40% of WT mice remained alive (Fig 2B). Viral titers were higher in infected *Prnp*^*0/0*^ lungs than in control WT lungs (Fig 2B). According to the Reed and Muench method [34], a 50% mouse lethal dose (MLD50) for IAV/PR8 was calculated as 66 IFU in WT mice and less than 50 IFU in *Prnp*^*0/0*^ mice. We also used *Prnp*^*0/0*^ and *Prnp*^*+/+*^ littermates for intranasal infection with 50 IFU of IAV/PR8. Male and female *Prnp*^*+/+*^ mice showed a mortality rate of about 40% at 14 dpi (Fig 2C). However, more than 80% of male and female *Prnp*^*0/0*^ mice died by 14 dpi (Fig 2C). Higher viral titers were observed in infected *Prnp*^*0/0*^ lungs compared to control *Prnp*^*+/+*^ lungs (Fig 2C).

We also tested other IAV strains, A/Aichi/2/68 (H3N2) and A/WSN/33 (H1N1) (hereafter referred to as IAV/Aichi and IAV/WSN, respectively), for their pathogenicity in *Prnp*^*0/0*^ mice. *Prnp*^*0/0*^ and WT mice were intranasally infected with 500 IFU of IAV/Aichi and 3,000 IFU of IAV/WSN. IAV/WSN belong to the same H1N1 subtype family as IAV/PR8. However, IAV/WSN was established by passages in mouse brains, thus being neurotropic, while IAV/PR8 is highly pathogenic to lungs [35]. Therefore, higher virus titers were used for intranasal infection with IAV/WSN. No male *Prnp*^*0/0*^ mice were alive by 14 dpi with IAV/Aichi and IAV/WSN (Fig 2D and 2E). However, about 90% and 75% of male WT mice survived at 14 dpi with IAV/Aichi and IAV/WSN, respectively (Fig 2D and 2E). Virus titers were higher in *Prnp*^*0/0*^ lungs than in WT lungs after infection with IAV/Aichi and IAV/WSN (Fig 2D and 2E). Higher mortality was also observed in female *Prnp*^*0/0*^ mice infected with IAV/Aichi and IAV/WSN, compared to control WT mice (Fig 2D and 2E). Taken together, these results indicate that *Prnp*^*0/0*^ mice are highly susceptible to IAV infection, with higher mortality and higher virus loads in the lungs compared to WT mice, suggesting that PrP^C^ could have a protective role against lethal infection with IAVs.

### Transgenic expression of PrP^C^ rescues *Prnp*^*0/0*^ mice from IAV infection

**Fig 3:**
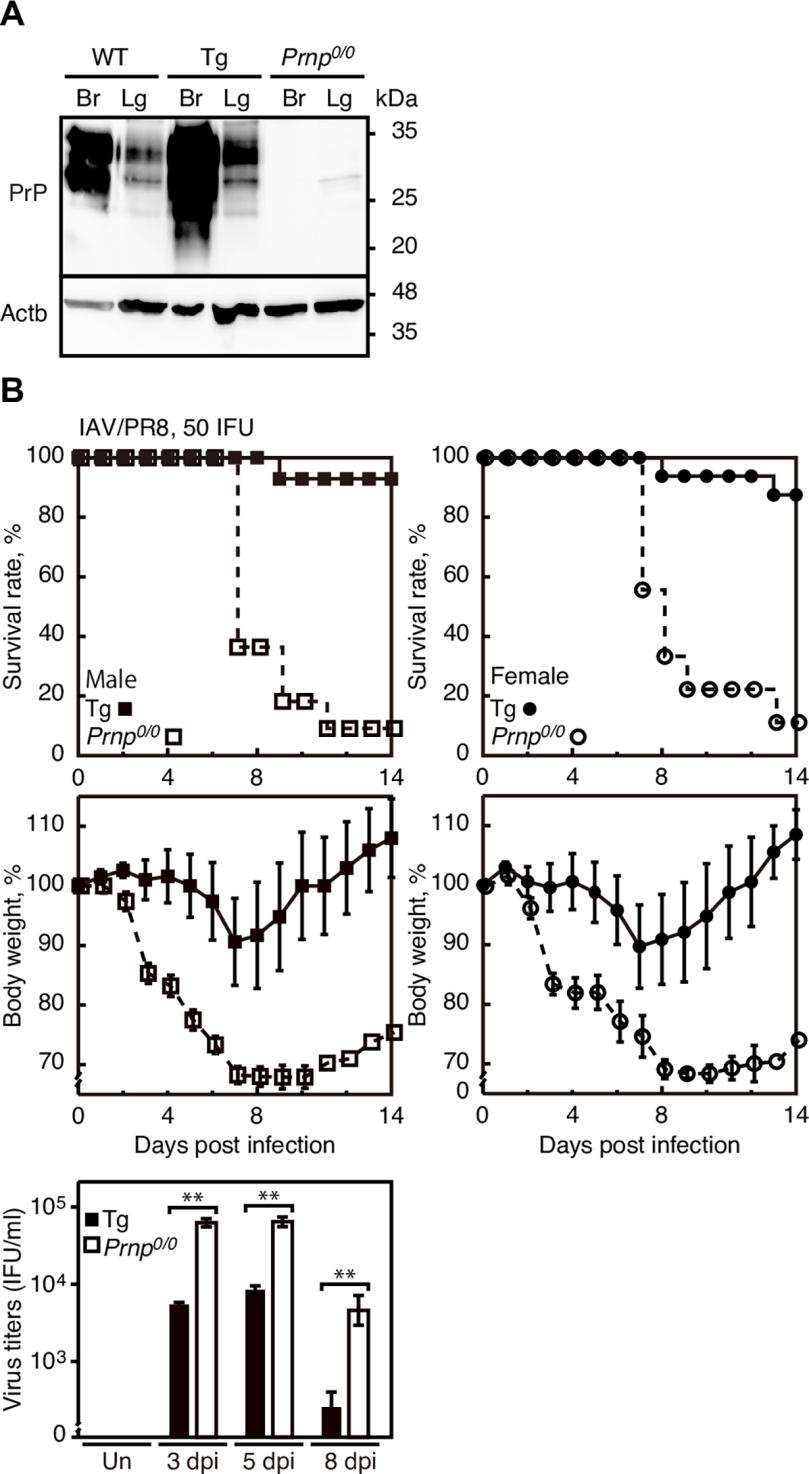
Transgenic expression of PrP^C^ rescues *Prnp*^*0/0*^ mice from lethal infection with IAVs. (A) Western blotting of the brains (Br) and lungs (Lg) of WT, Tg(MoPrP)/*Prnp*^*0/0*^, and *Prnp*^*0/0*^ mice with 6D11 anti-PrP antibody. Actb is an internal control. (B) Mortality and body weight of male (left panels) and female (right panels) *Prnp*^*0/0*^ and Tg(MoPrP)/*Prnp*^*0/0*^ mice after intranasal infection with 50 IFU of IAV/PR8 (male *Prnp*^*0/0*^, n = 11; male Tg(MoPrP)/*Prnp*^*0/0*^, n = 14; female *Prnp*^*0/0*^, n = 9; Tg(MoPrP)/*Prnp*^*0/0*^, n = 16). The *p* value for mortality: *p*<0.0001 in both genders. Lower panel: Viral titers in the lungs of male *Prnp*^*0/0*^ (n = 3) and Tg(MoPrP)/*Prnp*^*0/0*^ mice (n = 3) uninfected (Un) and infected with IAV/PR8 (50 IFU) at 3, 5, and 8 dpi. The 0 dpi in the graphs of survival rate and body weight is 5–10 min after infection. **, p<0.01. Error bars, SD.

To confirm that lack of PrP^C^ is responsible for the higher susceptibility of *Prnp*^*0/0*^ mice to IAV infection, 50 IFU of IAV/PR8 were intranasally infected into Tg(MoPrP)/*Prnp*^*0/0*^ mice, in which multiple copies of the transgene encoding mouse PrP^C^ are expressed on the *Prnp*^*0/0*^ background [36]. Western blotting showed higher expression of PrP^C^ in the lungs and brains of Tg(MoPrP)/*Prnp*^*0/0*^ mice than in WT mice (Fig 3A). Mortality was markedly reduced in male Tg(MoPrP)/*Prnp*^*0/0*^ mice compared to male *Prnp*^*0/0*^ littermates after infection (Fig 3B). More than 90% of male Tg(MoPrP)/*Prnp*^*0/0*^ mice survived the infection while only less than 10% of male *Prnp*^*0/0*^ littermates remained alive at 14 dpi. Virus titers were also reduced in Tg(MoPrP)/*Prnp*^*0/0*^ lungs compared to *Prnp*^*0/0*^ lungs (Fig 3B). A higher survival rate was also observed in female Tg(MoPrP)/*Prnp*^*0/0*^ mice after infection, compared to control female *Prnp*^*0/0*^ littermates (Fig 3B). These results confirm that the higher susceptibility of *Prnp*^*0/0*^ mice to IAV infection could result from the lack of PrP^C^.

### The OR region is important for PrP^C^ to protect against IAV infection

**Fig 4:**
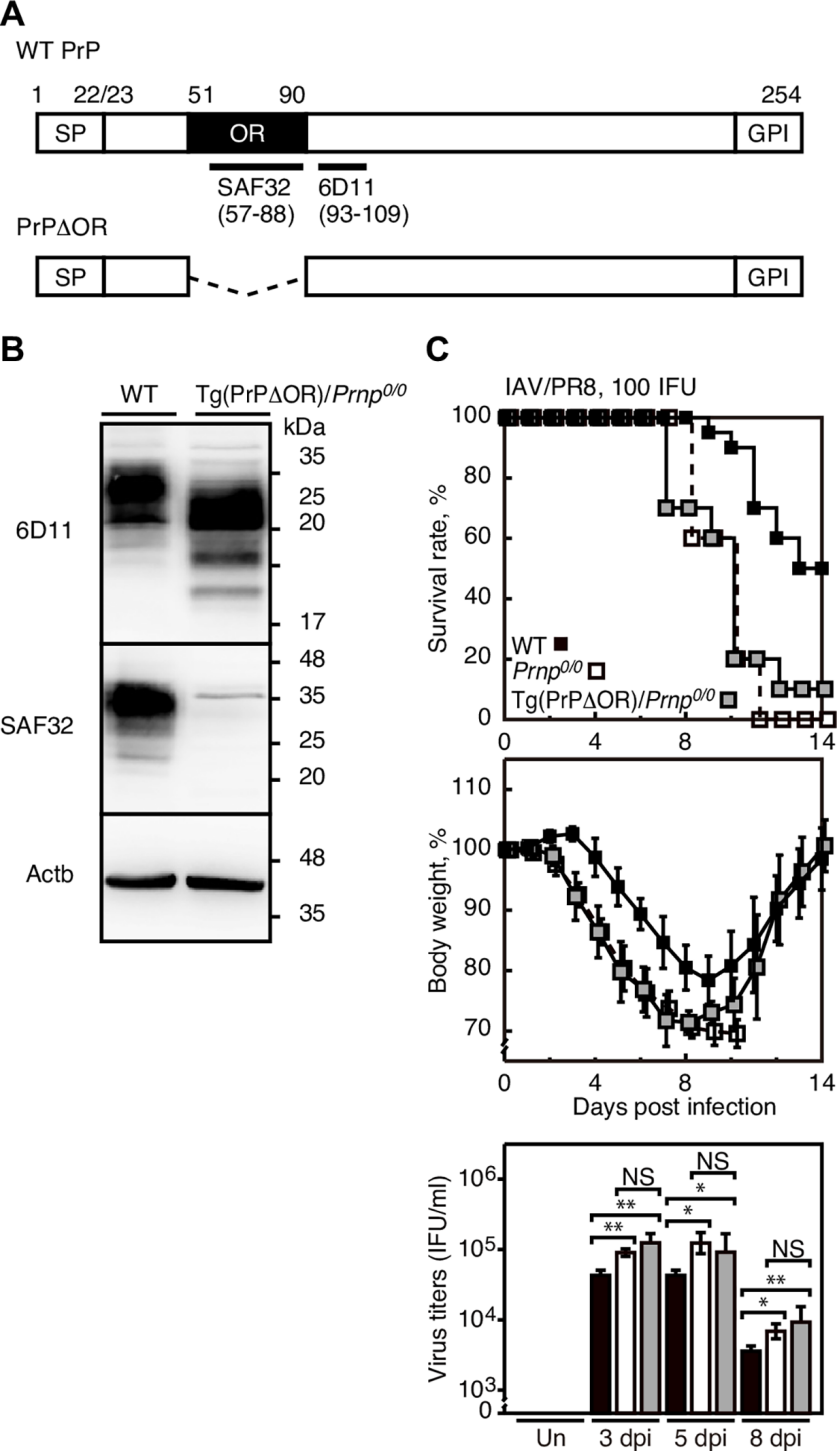
Transgenic expression of PrPΔOR fails to rescue *Prnp*^*0/0*^ mice from lethal infection with IAVs. (A) Schematic diagrams of the protein structure for WT PrP and PrPΔOR with the epitopes of SAF32 and 6D11 anti-PrP antibodies. SP, signal peptide; GPI, GPI anchor signal. (B) Western blotting of WT and Tg(PrPΔOR)/*Prnp*^*0/0*^ lungs with 6D11 and SAF32 anti-PrP antibodies. Actb is an internal control. (C) Mortality and body weight of male WT (n = 10), Tg(PrPΔOR)/*Prnp*^*0/0*^ (n = 10), and *Prnp*^*0/0*^ (n = 5) mice after intranasal infection with 100 IFU of IAV/PR8. The *p* value for mortality: WT vs *Prnp*^*0/0*^ mice, *p* = 0.0011; WT vs Tg(PrPΔOR)/*Prnp*^*0/0*^ mice, *p* = 0.009; Tg(PrPΔOR)/*Prnp*^*0/0*^ vs *Prnp*^*0/0*^ mice, *p* = 0.7022. Lower panel: Viral titers in the lungs of male *Prnp*^*0/0*^ (n = 3) and Tg(MoPrP)/*Prnp*^*0/0*^ mice (n = 3) uninfected (Un) and infected with IAV/PR8 (100 IFU) at 3, 5, and 8 dpi. The 0 dpi in the graphs of survival rate and body weight is 5–10 min after infection. *, p<0.05; **, p<0.01. NS, not significant. Error bars, SD.

We then investigated whether the OR region might be involved in the protective role of PrP^C^ against lethal infection with IAVs, by intranasal infection with 100 IFU of IAV/PR8 into Tg(PrPΔOR)/*Prnp*^*0/0*^ mice and their *Prnp*^*0/0*^ littermates. Tg(PrPΔOR)/*Prnp*^*0/0*^ mice express transgenic mouse PrP with a deletion of the OR region alone on the *Prnp*^*0/0*^ background [37]. Western blotting with 6D11 anti-PrP antibody, which recognizes residues 93–109 of mouse PrP, revealed expression of PrPΔOR in Tg(PrPΔOR)/*Prnp*^*0/0*^ lungs and PrP^C^ in WT lungs (Fig 4A and 4B). SAF32 anti-PrP antibody, which recognizes the OR region, did not detect PrPΔOR in Tg(PrPΔOR)/*Prnp*^*0/0*^ lungs (Fig 4A and 4B), confirming lack of the OR region in PrPΔOR. We increased the dose of IAV/PR8 for infection into Tg(PrPΔOR)/*Prnp*^*0/0*^ mice to 100 IFU since they were highly resistant to 50 IFU of IAV/PR8. IAV/PR8 infection caused similar mortality in Tg(PrPΔOR)/*Prnp*^*0/0*^ and *Prnp*^*0/0*^ mice (Fig 4C). However, mortality in these mice was significantly higher than that in control WT mice (Fig 4C). Only 10% of Tg(PrPΔOR)/*Prnp*^*0/0*^ mice and no *Prnp*^*0/0*^ mice survived the infection while 50% of WT mice were alive at 14 dpi (Fig 4C). Tg(PrPΔOR)/*Prnp*^*0/0*^ and *Prnp*^*0/0*^ lungs showed similar virus titers, but they were higher than those in WT lungs (Fig 4C). These results suggest that the OR region could play an important role for PrP^C^ to protect against lethal infection with IAVs in mice.

### More severe inflammation in IAV-infected *Prnp*^*0/0*^ lungs

**Fig 5:**
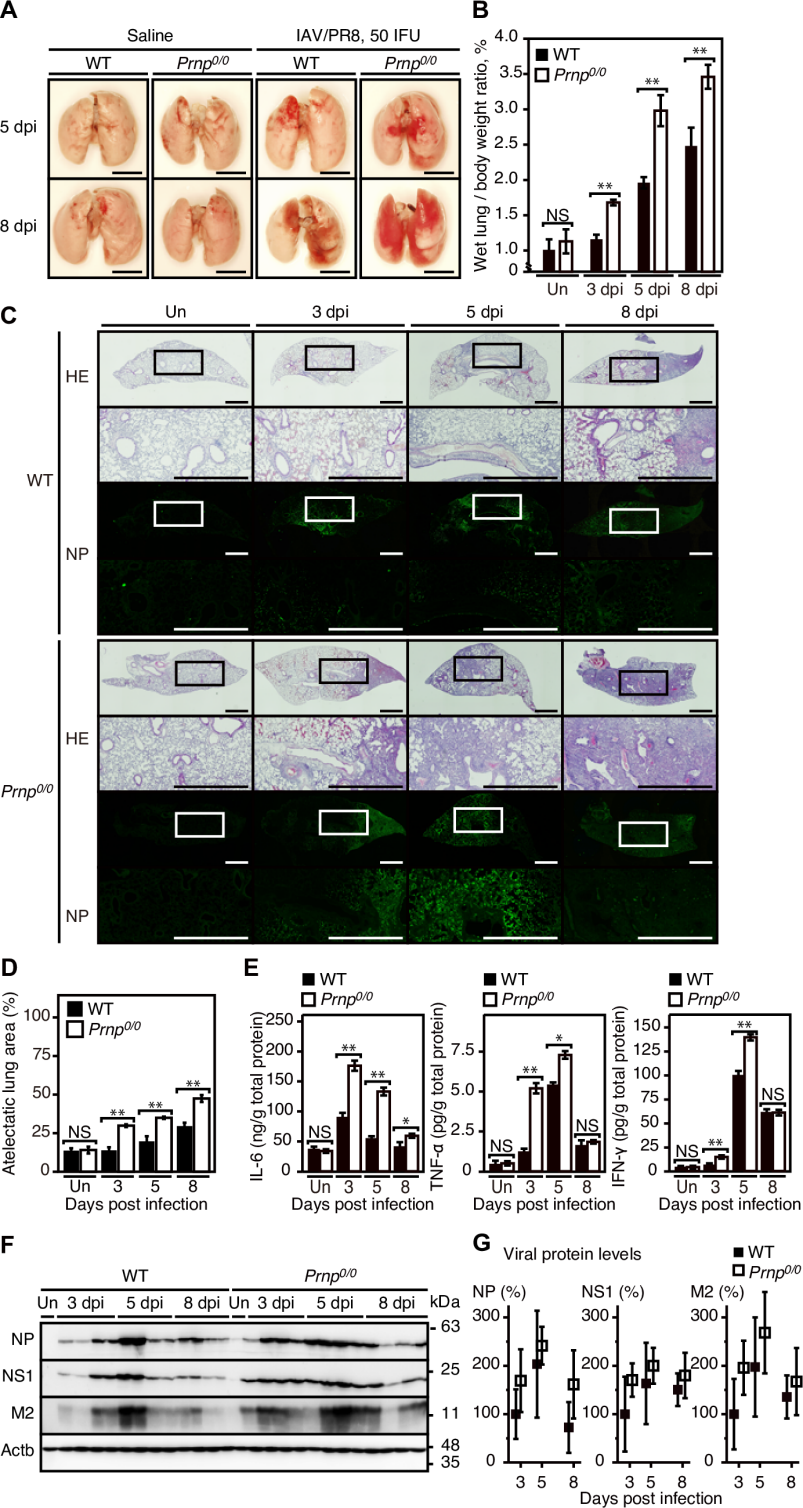
Pulmonary inflammation and viral loads are higher in IAV-infected *Prnp*^*0/0*^ lungs. (A) Macroscopic pictures of the lungs of WT and *Prnp*^*0/0*^ mice administrated with saline as uninfected control or infected with IAV/PR8 (50 IFU) at 5 and 8 dpi. Bar, 1 cm. (B) Wet lung weights of WT and *Prnp*^*0/0*^ mice (n = 3) uninfected (Un) and infected with 50 IFU of IAV/PR8 at 3, 5, and 8 dpi. (C) Hemotoxylin-Eosin staining of the lungs of WT and *Prnp*^*0/0*^ mice uninfected (Un) and infected with 50 IFU of IAV/PR8 at 3, 5, and 8 dpi and immunofluorescent staining for the viral protein NP in the consecutive lung sections. Bar, 200 μm. Insets show 3 times-magnified images of the squares. (D) Atelectatic area in the lungs of WT (n = 3) and *Prnp*^*0/0*^ mice (n = 3) uninfected (Un) and infected with 50 IFU of IAV/PR8 at 3, 5, and 8 dpi. (E) Levels of pro-inflammatory cytokines, IL-6, TNF-α, and IFN-γ, in the lungs of WT (n = 3) and *Prnp*^*0/0*^ mice (n = 3) at uninfected (Un) and infected with 50 IFU of IAV/PR8 at 3, 5, and 8 dpi. (F) Western blotting of the lungs of WT and *Prnp*^*0/0*^ mice uninfected (Un) and infected with 50 IFU of IAV/PR8 at 3, 5, and 8 dpi for viral proteins, NP, NS1, and M2. Actb is an internal control. (G) Quantification of the intensity for each viral protein in (F) after normalization against β-actin. Signal intensity of the proteins in the lungs was evaluated against that in WT lungs at 3 dpi. *, p<0.05; **, p<0.01. Error bars, SD.

To gain insights into the protective role of PrP^C^ against lethal infection with IAVs, we investigated the pathology of IAV/PR8 (50 IFU)-infected *Prnp*^*0/0*^ and WT lungs. No macroscopic lesions were observed on the lung surface of control saline-administrated *Prnp*^*0/0*^ and WT mice (Fig 5A). In contrast, reddish lesions were evident on the surface of infected WT and *Prnp*^*0/0*^ lungs at 5 and 8 dpi, with larger size and higher number of the lesions in the *Prnp*^*0/0*^ lungs than in the WT lungs (Fig 5A). *Prnp*^*0/0*^ and WT lungs had increased wet weights after infection, with the *Prnp*^*0/0*^ lungs being significantly heavier than the WT lungs (Fig 5B), suggesting higher exudates in *Prnp*^*0/0*^ lungs than in WT lungs after infection. Microscopic examinations showed higher infiltration of inflammatory cells in *Prnp*^*0/0*^ lungs than in WT lungs after infection (Fig 5C). Immunofluorescent staining also showed viral nucleocapsid protein NP accumulated in the inflammatory regions (Fig 5C). Atelectatic areas were therefore larger in *Prnp*^*0/0*^ lungs than in WT lungs after infection (Fig 5D). We also investigated levels of inflammatory cytokines, including interleukin-6 (IL-6), tumor necrosis factor-α (TNF-α), and interferon-γ (IFN-γ), in these infected lungs. All the cytokines examined had higher levels in *Prnp*^*0/0*^ lungs than in WT lungs (Fig 5E). We also investigated viral proteins in these infected lungs. Western blotting showed that viral proteins, including NP, NS1 nonstructural protein, and M2 matrix protein, became detectable in *Prnp*^*0/0*^ and WT lungs at 3 dpi, reached a peak level at 5 dpi, and decreased at 8 dpi (Fig 5F), with slightly but not significantly higher levels in the *Prnp*^*0/0*^ lungs than in the WT lungs (Fig 5G).

### Active immune responses against IAV infection in *Prnp*^*0/0*^ mice

We evaluated innate and adaptive immune responses against IAV infection in *Prnp*^*0/0*^ mice. To this end, we first investigated expression of the innate immunity-related genes, including those for retinoic acid-inducible gene I (RIG-I), melanoma differentiation-associated protein 5 (MDA5), TNF-α, IFN-α and IFN-γ, in IAV/PR8 (50 IFU)-infected WT and *Prnp*^*0/0*^ lungs. Reverse transcriptase-polymerase chain reaction (RT-PCR) showed upregulated expression of these genes in infected WT and *Prnp*^*0/0*^ lungs (S2A Fig). However, expression levels of these genes except for the MDA5 gene were higher in infected *Prnp*^*0/0*^ lungs than in control WT lungs (S2A Fig). Expression of the viral NP gene was also higher in infected *Prnp*^*0/0*^ lungs than in control WT lungs (S2A Fig), suggesting that the higher expression of the innate immune-related genes in infected *Prnp*^*0/0*^ lungs might be due to the higher viral loads in the lungs. We then investigated antibody responses against IAV/PR8 infection in WT and *Prnp*^*0/0*^ mice. Plasma levels of IAV/PR8-specific IgG and IgM antibodies were similarly elevated in infected WT and *Prnp*^*0/0*^ mice (S2B Fig). Enzyme-linked immunoSpot (ELISPOT) assay also showed that the spot number of TNF-α- or IFN-γ-secreting cells was the same in the lungs and spleens of infected *Prnp*^*0/0*^ and WT mice (S2C Fig). These results indicate that *Prnp*^*0/0*^ mice could activate innate and adaptive immune responses against IAV infection.

### Higher epithelial cell damage in IAV-infected *Prnp*^*0/0*^ lungs

**Fig 6:**
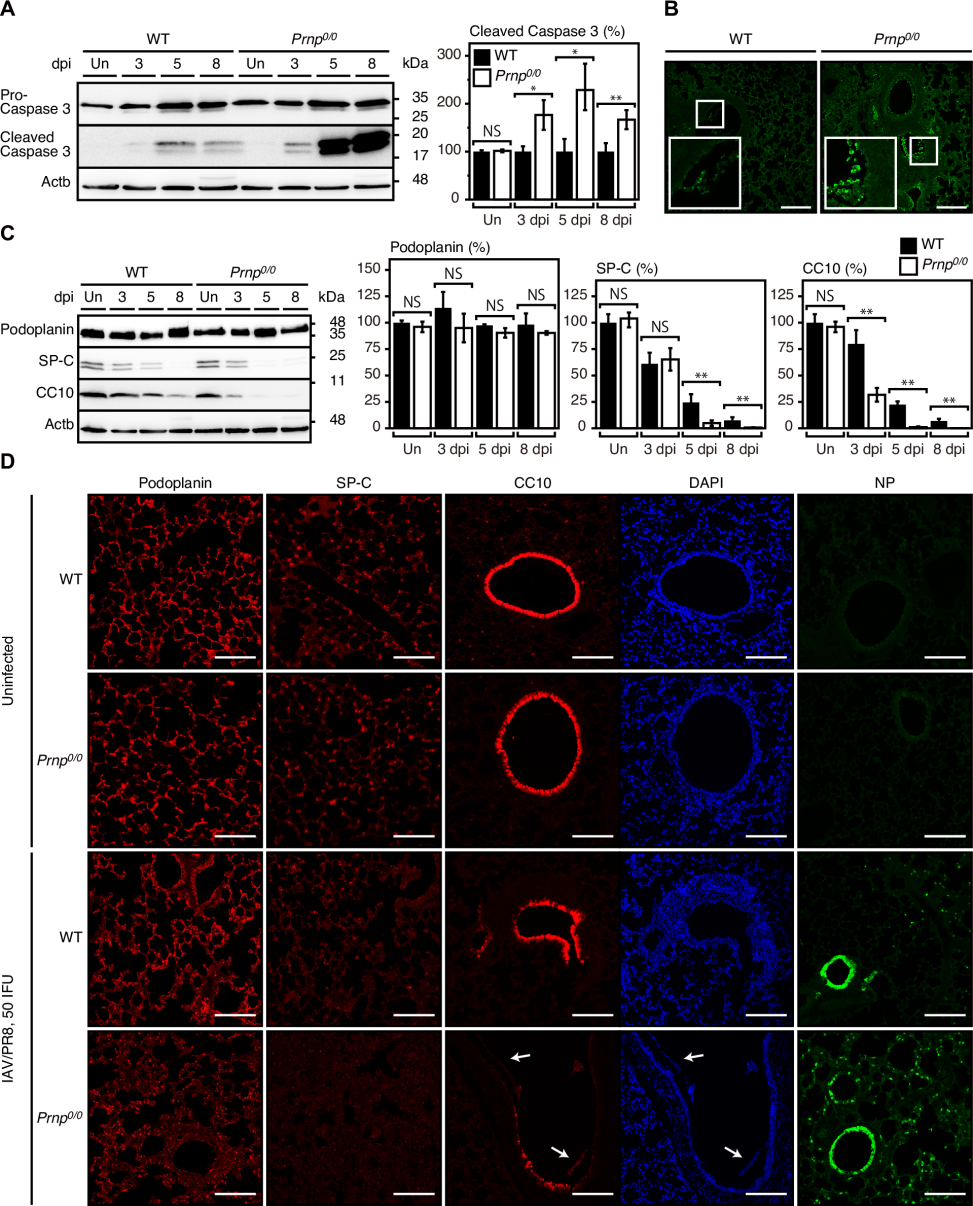
Epithelial cells are highly apoptotic in IAV-infected *Prnp*^*0/0*^ lungs. (A) Left panel: Western blotting of the lungs of WT and *Prnp*^*0/0*^ mice uninfected (Un) and infected with 50 IFU of IAV/PR8 at 3, 5, and 8 dpi for pro-caspase 3 and its cleaved fragments. Actb is an internal control. Right panel: Quantification of the cleaved fragments of caspase 3 after normalization against β-actin. Signal intensity of the cleaved fragments of caspase 3 in *Prnp*^*0/0*^ lungs (n = 3) was evaluated against that in WT lungs (n = 3). (B) TUNEL staining of IAV/PR8 (50 IFU)-infected WT and *Prnp*^*0/0*^ lungs at 5 dpi. Bar, 400 μm. Insets show 2.5 times-magnified images of the squares. (C) Left panel: Western blotting of the lungs of WT and *Prnp*^*0/0*^ mice uninfected (Un) and infected with 50 IFU of IAV/PR8 at 3, 5, and 8 dpi for podoplanin, SP-C, and CC10. Actb is an internal control. Right panels: Quantification of podoplanin, SP-C, and CC10 after normalization against β-actin (n = 3 for each mouse group). Signal intensity for podoplanin, SP-C, and CC10 in *Prnp*^*0/0*^ lungs was evaluated against that in uninfected WT lungs. (D) Immunofluorescence staining of uninfected and IAV/PR8 (50 IFU)-infected WT and *Prnp*^*0/0*^ lungs at 5 dpi with antibodies against podoplanin, SP-C and CC10, and with DAPI. Right panels: Immunofluorescent staining for the viral protein NP in the lungs of these mice. Bar, 400 μm. NS, not significant; *, p<0.05; **, p<0.01. Error bars, SD.

To understand the protective mechanism of PrP^C^ against lethal infection with IAVs, we investigated apoptotic cell death in IAV/PR8 (50 IFU)-infected *Prnp*^*0/0*^ and WT lungs, by performing Western blotting for the cleaved fragments of the apoptotic marker caspase 3. *Prnp*^*0/0*^ and WT lungs showed increased the fragments after infection (Fig 6A). However, the fragments were higher in infected *Prnp*^*0/0*^ lungs than in control WT lungs (Fig 6A). Terminal deoxynucleotidyl transferase-mediated dUTP nick-end labeling (TUNEL) staining also showed more abundant apoptotic cells in alveolar and bronchiolar epithelial areas in *Prnp*^*0/0*^ lungs than in WT lungs (Fig 6B). We also performed Western blotting with anti-podoplanin, anti-SP-C, and anti-CC10 antibodies. Podoplanin levels were unaffected in *Prnp*^*0/0*^ and WT lungs after infection (Fig 6C). This is consistent with IAV/PR8 infection not damaging AT1 cells in C57BL/6 mice [38]. In contrast, SP-C and CC10 were markedly decreased in *Prnp*^*0/0*^ and WT lungs after infection, with their levels significantly lower in infected *Prnp*^*0/0*^ lungs than in control WT lungs (Fig 6C). Consistently, immunofluorescence staining showed that SP-C-positive AT2 cells and CC10-positive Clara cells were less in infected *Prnp*^*0/0*^ lungs than in control WT lungs, while podoplanin-positive AT1 cells were similarly observed in infected these lungs (Fig 6D). These results suggest that AT2 and Clara cells in *Prnp*^*0/0*^ lungs could be more vulnerable to apoptosis than those in WT lungs after infection with IAVs, and that PrP^C^ could exert an anti-apoptotic activity in AT2 and Clara cells after infection with IAVs.

### Anti-oxidant rescues *Prnp*^*0/0*^ mice from lethal infection with IAV

**Fig 7:**
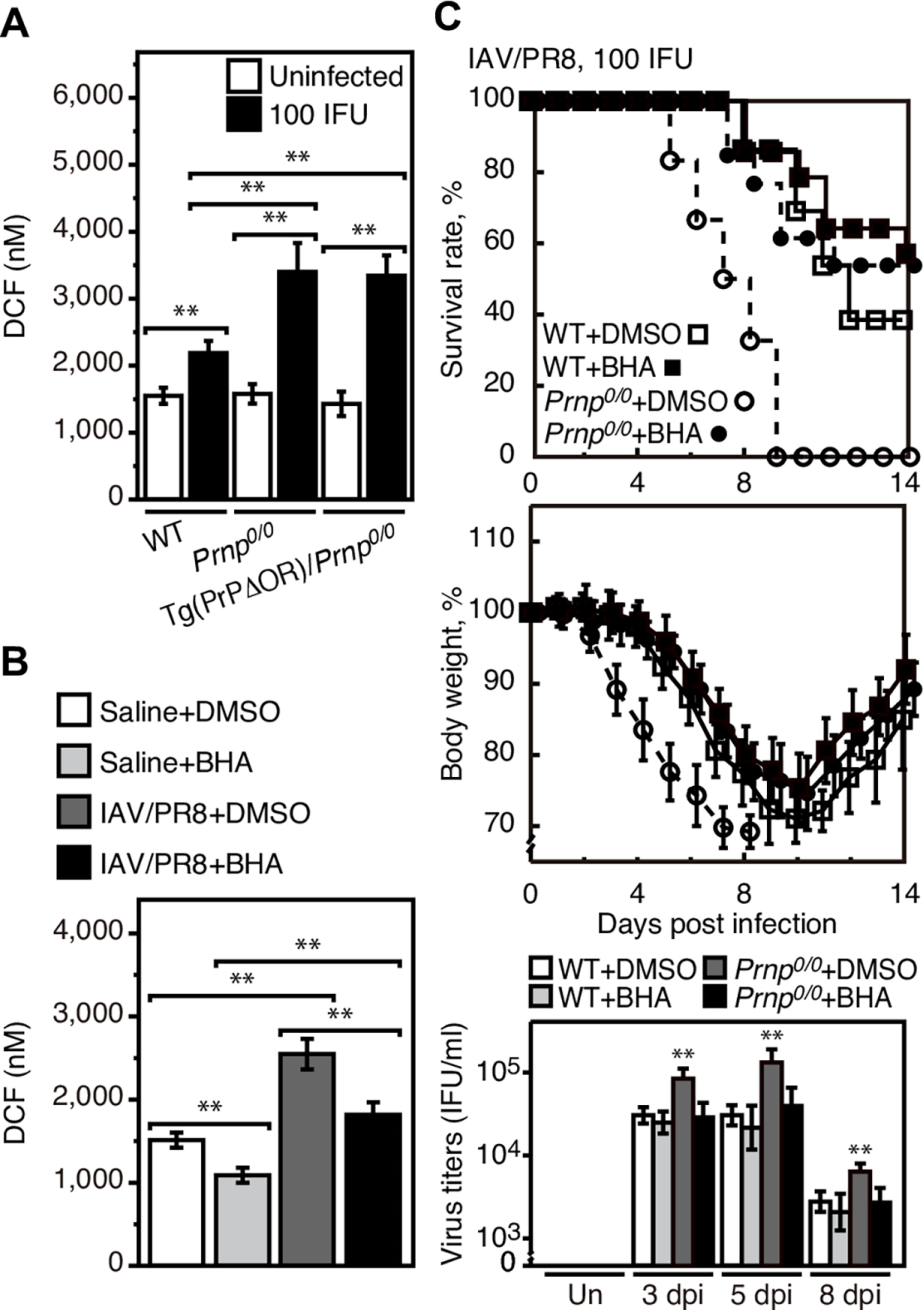
BHA rescue *Prnp*^*0/0*^ mice from lethal infection with IAV. (A) DCF levels representing ROS levels in the lungs of WT, *Prnp*^*0/0*^, and Tg(PrPΔOR)/*Prnp*^*0/0*^ mice uninfected and infected with IAV/PR8 (100 IFU) (n = 3 in each mouse group) at 5 dpi. (B) DCF levels in the lungs of WT mice administrated with saline as a control and infected with IAV/PR8 (100 IFU) at 5 dpi after treatment with BHA or DMSO (n = 3 in each group). (C) Mortality and body weight of BHA-treated WT (n = 14) and *Prnp*^*0/0*^ (n = 13) mice and BHA-untreated WT (n = 13) and *Prnp*^*0/0*^ (n = 12) mice after intranasal infection with 100 IFU of IAV/PR8. The *p* value for mortality: untreated vs treated WT mice, *p* = 0.3603; untreated vs treated *Prnp*^*0/0*^ mice, *p* = 0.0005; untreated WT vs *Prnp*^*0/0*^ mice, *p*<0.0001; treated WT vs treated *Prnp*^*0/0*^ mice, *p* = 0.6706. Lower panel: Viral titers in the lungs of each group of mice uninfected (Un) and infected with IAV/PR8 (100 IFU) at 3, 5, and 8 dpi (n = 3 in each mouse group). The 0 dpi in the graphs of survival rate and body weight is 5–10 min after infection. **, p<0.01; NS, not significant. Error bars, SD.

To investigate the role of ROS in the higher mortality of IAVs-infected *Prnp*^*0/0*^ mice, we addressed whether *Prnp*^*0/0*^ mice could be rescued from lethal infection with IAV/PR8 by treatment with a ROS scavenger. To this end, we first measured ROS in the lungs of *Prnp*^*0/0*^ and WT mice intranasally infected with IAV/PR8 (100 IFU). The virus dose used was higher than 1 MLD50 since it is assumed that the effect of a ROS scavenger on the survival rate of infected mice, if any, would be evaluated more easily for the mice developing mortality more than 50% after infection than those with less than 50% mortality after infection. No difference in ROS levels was detected between uninfected *Prnp*^*0/0*^ and WT lungs (Fig 7A). IAV/PR8 infection at 5 dpi increased ROS levels in *Prnp*^*0/0*^ and WT lungs (Fig 7A). However, ROS levels were higher in *Prnp*^*0/0*^ lungs than in control WT lungs (Fig 7A). ROS levels were also higher in infected Tg(PrPΔOR)/*Prnp*^*0/0*^ lungs than in control WT lungs at 5 dpi (Fig 7A). These results suggest that PrP^C^ might exert an anti-oxidative activity to reduce ROS levels through the OR region in IAV-infected lungs. We then examined the anti-oxidative effect of butylated hydroxyanisole (BHA), a ROS scavenger, on ROS levels in the lungs of IAV/PR8 (100 IFU)-infected WT mice. Treatment with BHA for 4 days starting at 2 dpi effectively reduced ROS in infected WT lungs at 5 dpi (Fig 7B). We then similarly treated *Prnp*^*0/0*^ and WT mice with BHA after intranasal infection with IAV/PR8 (100 IFU). The treatment decreased the mortality of infected WT mice (Fig 7C). This could be consistent with that ROS could be a major player in IAV infection-induced lung injury [30–33]. The mortality of infected *Prnp*^*0/0*^ mice was also decreased to that of control WT mice after treatment with BHA (Fig 7C). Viral titers were also decreased in *Prnp*^*0/0*^ lungs to those in WT lungs after treatment with BHA (Fig 7C). These results suggest that the higher ROS levels in *Prnp*^*0/0*^ lungs could be involved in the higher mortality of *Prnp*^*0/0*^ mice after infection with IAVs.

### Higher expression of xanthine oxidase (XO) and lower activity of SOD1 in IAV-infected *Prnp*^*0/0*^ lungs

**Fig 8:**
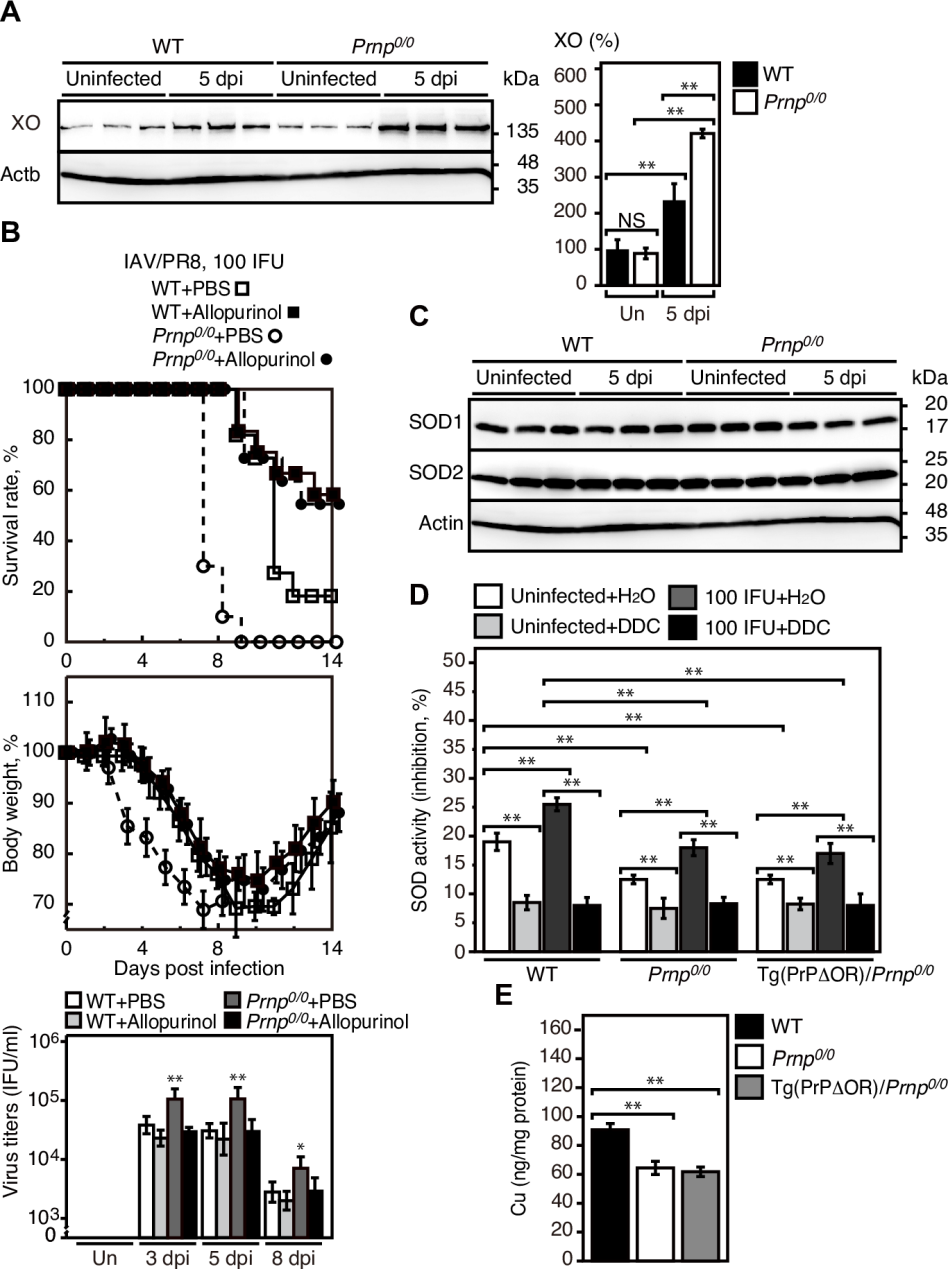
Higher expression of XO and lower activity of SOD1 in IAV-infected *Prnp*^*0/0*^ lungs. (A) Western blotting of the lungs of WT and *Prnp*^*0/0*^ mice uninfected (Un) and infected with IAV/PR8 (100 IFU) at 5 dpi for XO. Actb is an internal control. Right panel: Quantification of XO after normalization against β-actin. Signal intensity of XO in lungs was evaluated against that in uninfected WT lungs. (B) Mortality and body weight of allopurinol-treated WT (n = 24) and *Prnp*^*0/0*^ (n = 11) mice and of control PBS-treated WT (n = 11) and *Prnp*^*0/0*^ (n = 10) mice after intranasal infection with 100 IFU of IAV/PR8. The *p* value for mortality: PBS-treated vs allopurinol-treated WT mice, *p* = 0.0104; PBS-treated vs allopurinol-treated *Prnp*^*0/0*^ mice, *p* = 0.0005; PBS-treated WT vs *Prnp*^*0/0*^ mice, *p*<0.0001; allopurinol-treated WT vs *Prnp*^*0/0*^ mice, *p* = 0.0529. Right panel: Viral titers in the lungs of each group of mice uninfected (Un) or infected with IAV/PR8 (100 IFU) at 3, 5, and 8 dpi (n = 3 in each mouse group). (C) Western blotting of the lungs of WT and *Prnp*^*0/0*^ mice uninfected and infected with IAV/PR8 (100 IFU) at 5 dpi for SOD1 and 2. Actb is an internal control. (D) SOD activity in the lungs of WT, *Prnp*^*0/0*^, and Tg(PrPΔOR)/*Prnp*^*0/0*^ mice uninfected and infected with IAV/PR8 (100 IFU) after treatment with or without DDC (n = 3 for each mouse group). (E) Cu ion content in uninfected WT, *Prnp*^*0/0*^, and Tg(PrPΔOR)/*Prnp*^*0/0*^ lungs (n = 3 for each mouse group). The 0 dpi in the graphs of survival rate and body weight is 5–10 min after infection. Un, uninfected. **, p<0.01; NS, not significant. Error bars, SD.

To investigate the role of XO, a major ROS-generating enzyme in IAV-infected lungs [30], in IAV-infected *Prnp*^*0/0*^ lungs, we first perform Western blotting of IAV/PR8 (100 IFU)-infected *Prnp*^*0/0*^ and WT lungs for XO. Expression of XO was increased in these infected lungs (Fig 8A). However, the expression levels of XO were higher in infected *Prnp*^*0/0*^ lungs than in control WT lungs (Fig 8A). We then treated *Prnp*^*0/0*^ and WT mice with the XO inhibitor allopurinol after intranasal infection with IAV/PR8 at 100 IFU, a dose higher than 1 MLD50 in WT mice. Allopurinol treatment starting from one day before intranasal infection with IAV/PR8 to 14 dpi reduced the mortality of *Prnp*^*0/0*^ and WT mice to a similar rate (Fig 8B). Viral titers were also decreased in *Prnp*^*0/0*^ lungs compared to those in WT lungs after treatment with allopurinol (Fig 8B). These results suggest that XO could be a key ROS-generating enzyme in IAV-infected *Prnp*^*0/0*^ and WT lungs, and that the higher expression of XO in IAV-infected *Prnp*^*0/0*^ lungs could be involved in the higher mortality of IAVs-infected *Prnp*^*0/0*^ mice probably through producing higher levels of ROS.

We also investigated infected *Prnp*^*0/0*^ and WT lungs for the enzymatic activity of SOD, an anti-oxidative enzyme in IAV-infected lungs [31]. Western blotting revealed similar expression of SOD1 and SOD2 between uninfected and infected *Prnp*^*0/0*^ or WT lungs (Fig 8C). However, the total SOD activity was significantly lower in uninfected *Prnp*^*0/0*^ lungs than in uninfected WT lungs (Fig 8D). The SOD1-specific inhibitor diethyl-dithio-carbamate (DDC) reduced SOD activity in both uninfected *Prnp*^*0/0*^ and WT lungs to the same levels (Fig 8D), suggesting that SOD1 activity might be impaired in *Prnp*^*0/0*^ lungs. IAV/PR8 infection increased the total SOD activity in both WT and *Prnp*^*0/0*^ lungs (Fig 8D). However, the activity was lower in infected *Prnp*^*0/0*^ lungs than in control WT lungs (Fig 8D). DDC decreased the SOD activity in infected *Prnp*^*0/0*^ and WT lungs to the levels in uninfected *Prnp*^*0/0*^ lungs (Fig 8D). These results suggest that SOD1 might not be fully activated in *Prnp*^*0/0*^ lungs after IAV infection. Lower SOD1 activity was also detected in infected and uninfected Tg(PrPΔOR)/*Prnp*^*0/0*^ lungs than in control WT lungs (Fig 8D). Cu ions, which are important for SOD1 activity, were lower in uninfected *Prnp*^*0/0*^ and Tg(PrPΔOR)/*Prnp*^*0/0*^ lungs than in control WT lungs (Fig 8E). These results suggest that PrP^C^ might function to maintain Cu levels and thereby might regulate SOD1 activity through the Cu-binding OR region in lungs.

### Primary *Prnp*^*0/0*^ lung cells are susceptible to IAV infection

To further gain insights into the protective role of PrP^C^ in IAV infection, we infected primary lung cells from WT, *Prnp*^*0/0*^ and Tg(MoPrP)/*Prnp*^*0/0*^ mice with IAV/PR8 at 1.0 multiplicity of infection (MOI). *Prnp*^*0/0*^ cells were more vulnerable to the infection than WT cells (S3A Fig). In contrast, Tg(MoPrP)/*Prnp*^*0/0*^ cells were highly resistant to the infection (S3A Fig). Higher expression of viral proteins NP, HA and M2 was observed in *Prnp*^*0/0*^ cells than in WT cells at 2 dpi (S3B Fig). In contrast, their expression was lower in Tg(MoPrP)/*Prnp*^*0/0*^ cells than in WT cells (S3B Fig). Higher activation of caspase 3 was detected in infected *Prnp*^*0/0*^ cells than control WT cells (S3B Fig). In contrast, activation of caspase 3 was lower in infected Tg(MoPrP)/*Prnp*^*0/0*^ cells than in control WT cells (S3B Fig). siRNA-mediated knockdown of PrP^C^ in the A549 human lung epithelial cells also caused higher expression of NP, HA and M2 and higher activation of caspase 3 after infection with IAV/PR8 (S3C Fig). ROS levels were also higher in infected *Prnp*^*0/0*^ cells and lower in infected Tg(MoPrP)/*Prnp*^*0/0*^ cells compared to those in control WT cells (S3D Fig). In contrast, SOD activity was lower in *Prnp*^*0/0*^ cells and higher in Tg(MoPrP)/*Prnp*^*0/0*^ cells after infection with IAV/PR8 compared to that in infected WT cells (S3E Fig). These results are consistent with those from the *in vivo* experiments, suggesting that PrP^C^ might exert a protective activity against IAV infection in a cell-autonomous way.

### Lipopolysaccharide (LPS) induces similar injuries in *Prnp*^*0/0*^ and WT lungs

Intranasal administration of LPS is known to cause lung injuries in mice [39]. To investigate whether PrP^C^ might be also protective against LPS-induced lung injuries, we intranasally administrated LPS into *Prnp*^*0/0*^ and WT mice. No *Prnp*^*0/0*^ and WT mice died from the administration (S4A Fig). The body weight of *Prnp*^*0/0*^ and WT mice was similarly reduced by 3 days after administration and thereafter increased (S4A Fig). Caspase 3 was similarly activated in WT and *Prnp*^*0/0*^ lungs 24 h after administration with LPS (S4B Fig). ROS and inflammatory cytokines, including TNF-α and IFN-γ, were also similarly elevated between *Prnp*^*0/0*^ and WT lungs 24 h after administration with LPS (S4C Fig). These results suggest that PrP^C^ might have no protective activity against LPS-induced lung injuries.

## Discussion

In the present study, we showed that *Prnp*^*0/0*^ mice were highly susceptible to infection with IAVs, with markedly higher mortality, compared to control WT mice. Pathological changes were more severe, inflammatory cytokines including IL-6, TNF-α, and IFN-γ were higher, and viral loads were higher in IAV/PR8-infected *Prnp*^*0/0*^ lungs. We confirmed that the higher mortality of infected *Prnp*^*0/0*^ mice is due to lack of PrP^C^, by demonstrating that transgenic expression of mouse PrP^C^ rescued *Prnp*^*0/0*^ mice from lethal infection with IAV/PR8. We also showed that mouse PrP lacking the OR region failed to protect *Prnp*^*0/0*^ mice from the lethal infection with IAV/PR8. These results suggest that PrP^C^ could have a protective role against lethal infection with IAVs through the OR region in mice.

*Prnp*^*0/0*^ mice activated innate and adaptive immune responses against IAV infection. These results rule out the possibility that lack of PrP^C^ might cause defective immune responses against IAV infection, therefore *Prnp*^*0/0*^ mice being highly susceptible to IAV infection. PrP^C^ was expressed in alveolar AT1 and 2 epithelial cells and bronchiolar Clara epithelial cells in lungs. Other investigators also reported expression of PrP^C^ in alveolar walls and Clara cells [8,40]. Consistent with the previous report showing that AT1 cells were resistant to infection with IAV/PR8 in WT mice [38], AT1 cells were unaffected by infection with IAV/PR8 not only in WT lungs but also in *Prnp*^*0/0*^ lungs. In contrast, infection with IAV/PR8 markedly damaged AT2 and Clara cells in *Prnp*^*0/0*^ and WT lungs. However, these epithelial cells in *Prnp*^*0/0*^ lungs were more susceptible to the infection than those in WT lungs. Caspase 3 was activated more robustly in *Prnp*^*0/0*^ lungs than in WT lungs after infection with IAV/PR8. TUNEL staining also displayed more abundant apoptotic cells in the alveolar and bronchiolar epithelial areas of infected *Prnp*^*0/0*^ lungs than in control WT lungs. Primary *Prnp*^*0/0*^ lung culture cells were also vulnerable to IAV/PR8 infection-induced apoptosis compared to control WT lung cells. These results suggest that AT2 and Clara epithelial cells in *Prnp*^*0/0*^ lungs might be more vulnerable to IAV infection-induced apoptosis than those in WT lungs, and that PrP^C^ might play an anti-apoptotic role in these lung epithelial cells in a cell-autonomous way. However, intranasal administration with LPS, which induces lung injuries through binding to Toll-like receptor 4 (TLR4) [41], similarly activated caspase 3 in *Prnp*^*0/0*^ and WT lungs, suggesting that the anti-apoptotic activity of PrP^C^ has no effect on the LPS/TLR4-induced apoptosis in lungs.

Viral loads were significantly but only slightly higher in *Prnp*^*0/0*^ lungs than in WT lungs after infection with IAV/PR8. It has been shown that caspase 3 activation induces efficient replication of IAV in cells [42]. It is thus possible that the slightly higher viral loads in IAV/PR8-infected *Prnp*^*0/0*^ lungs might be associated with the higher activation of caspase 3 observed in the lungs. Thus, PrP^C^ might exert its protective activity against IAV infection through its anti-apoptotic activity in lung epithelial cells, not through directly affecting viral replication efficiency in lungs. However, the possibility remains unanswered if PrP^C^ could directly affect IAV replication in the lungs, thereby reducing viral loads and eventually repressing caspase 3 activation in the lungs.

AT2 cells are small cuboidal cells covering about 2–5% of the alveolar surface area and secreting surfactant proteins, which are important to reduce alveolar surface tension [43,44]. Clara cells are the predominant cell type in bronchioles and known as important progenitor cells for the repair of bronchiolar epithelia [45]. Recently, it was reported that Clara cells are also major progenitor cells for alveolar epithelial regeneration through differentiation to AT1 and 2 alveolar cells after IAV infection [46,47]. AT2 and Clara cells were more severely damaged in *Prnp*^*0/0*^ lungs than in WT lungs after infection with IAV/PR8. It is thus possible that the AT2 cells-mediated regulation of alveolar surface tension and the Clara cells-mediated alveolar and bronchiolar epithelia regeneration after IAV infection might be disturbed more severely in *Prnp*^*0/0*^ lungs than in WT lungs after infection with IAVs, eventually causing higher mortality in *Prnp*^*0/0*^ mice infected with IAVs.

We showed that ROS levels were higher in IAV/PR8-infected *Prnp*^*0/0*^ lungs than in control WT lungs. We also showed that the ROS scavenger BHA rescued *Prnp*^*0/0*^ mice from lethal infection with IAV/PR8, reducing mortality to the levels in IAV/PR8-infected, BHA-treated control WT mice, suggesting that the higher ROS levels in infected *Prnp*^*0/0*^ lungs could be responsible for the higher mortality of *Prnp*^*0/0*^ mice infected with IAVs. It has been shown that *Prnp*^*0/0*^ cells were more susceptible to treatment with agents inducing oxidative stress, readily succumbing to apoptosis, compared with WT cells [23,48], suggesting that PrP^C^ could have a protective role against oxidative stress-induced apoptosis. Therefore, PrP^C^ might play an anti-oxidative role in lungs after infection with IAVs, thereby reducing ROS levels and protecting lung epithelial cells from IAV infection-induced apoptosis. We also demonstrated higher ROS levels in Tg(PrPΔOR)/*Prnp*^*0/0*^ lungs than in WT lungs after infection with IAV/PR8, suggesting that the OR region could be important for PrP^C^ to exert the anti-oxidative activity in lungs after infection with IAVs.

XO was shown to be a major ROS-generating enzyme in IAV-infected lungs [30]. We showed that XO expression was elevated in infected *Prnp*^*0/0*^ lungs compared to control WT lungs. We also showed that the XO inhibitor allopurinol rescued *Prnp*^*0/0*^ mice from lethal infection with IAV/PR8. These results suggest that the XO up-regulation observed in infected *Prnp*^*0/0*^ lungs might be responsible for the higher mortality in *Prnp*^*0/0*^ mice infected with IAVs. It has been shown that inflammatory cytokines such as TNF-α and IFN-γ up-regulate the expression of XO [49–51]. Higher levels of these cytokines were detected in infected *Prnp*^*0/0*^ lungs than in control WT lungs, suggesting that the higher expression of XO in infected *Prnp*^*0/0*^ lungs might be induced by the higher levels of these cytokines in the lungs.

We also showed that IAV/PR8 infection increased SOD1 activity in *Prnp*^*0/0*^ and WT lungs. However, SOD1 was not fully activated in infected *Prnp*^*0/0*^ lungs compared to control WT lungs, suggesting that SOD1 activation might be disturbed in *Prnp*^*0/0*^ lungs infected with IAVs. Together with the reported results that administration of pyran polymer-conjugated SOD1 successfully reduced the mortality of WT mice infected with IAV [31], it is suggested that the lower activity of SOD1 in infected *Prnp*^*0/0*^ lungs might be responsible for the higher mortality of *Prnp*^*0/0*^ mice infected with IAVs. Cu ions are important for the SOD1 enzymatic activity. We found that Cu ion content were lower in *Prnp*^*0/0*^ lungs than in WT lungs, suggesting that the lower activity of SOD1 in *Prnp*^*0/0*^ lungs might be due to the lower Cu content in the lungs. Reduced SOD1 activity and lower Cu content were also detected in Tg(PrPΔOR)/*Prnp*^*0/0*^ lungs. It is thus possible that PrP^C^ might have a role to maintain Cu levels in lungs through the OR region, thereby regulating SOD1 activity and eventually exerting an anti-oxidative activity in lungs. PrP^C^ is known to bind to Cu ions via the OR region, suggesting that PrP^C^ might transfer the bound Cu ions to and activate SOD1 [23,24]. However, the exact mechanism of how PrP^C^ is involved in the activation of SOD1 remains to be determined. It has been also proposed that PrP^C^ itself could have SOD activity [52]. However, other investigators failed to confirm this proposed SOD activity in PrP^C^ [53,54]. Elucidation of the mechanism underlying the anti-oxidative function of PrP^C^ could be helpful for further understanding the pathogenesis of IAV infection and for development of anti-influenza therapeutics based on the PrP^C^-mediated protective mechanism.

Anti-oxidative therapeutics against IAV infection, by targeting the ROS-generating enzymes or by administrating anti-oxidants or anti-oxidant enzymes, has been shown to successfully protect mice from lethal infection with IAVs [30–33]. Our current results showing that PrP^C^ could have a protective role against lethal infection with IAVs in mice possible by exerting ant-oxidative activity, suggest PrP^C^ to be a new target molecule for anti-oxidative therapeutics against IAV infection. It has been reported that PrP^C^ elicited a protective signal against anisomycin-induced apoptosis in neurons via interaction with stress-inducible protein 1 (STI1), a STI1-derived peptide, or anti-PrP antibodies [55,56], and that the interaction with STI1 could be involved in PrP^C^-dependent activation of SOD [57]. It is thus interesting to investigate whether these ligands could elicit the protective activity of PrP^C^ against IAV infection.

## Materials and methods

### Ethics statement

All animal experiments were conducted in compliance with Japanese legislation (Act on Welfare and Management of Animals). The Ethics Committee of Animal Care and Experimentation of Tokushima University approved the animal experiments in this study (approval number T27-86). Animals were cared for in accordance with The Guiding Principle for Animal Care and Experimentation of Tokushima University and guidelines under the jurisdiction of the Ministry of Education, Culture, Sports, Science and Technology, Japan.

### Animals

C57BL/6 mice were purchased from Japan SLC Inc. (Shizuoka, Japan). *Prnp*^*0/0*^ mice used in this study had been obtained elsewhere by at least more than 9 time-backcrosses to C57BL/6 mice with *Prnp*^*0/0*^ mice, which originally carry a mixed background of C57BL/6×129Sv×FVB mice [37,58]. The backcrossed *Prnp*^*0/0*^ mice were maintained by intercrossing the backcrossed *Prnp*^*0/0*^ mouse pairs and used in this study. *Prnp*^*0/0*^ and *Prnp*^*+/+*^ littermates used in this study were produced by intercross of the backcrossed *Prnp*^*0/+*^ mouse pairs, which were produced by intercrossing the backcrossed *Prnp*^*0/0*^ mice with C57BL/6 mice. Tg(MoPrP)/*Prnp*^*0/0*^ mice were obtained elsewhere by intercross between the backcrossed *Prnp*^*0/0*^ mice and Tg(MoPrP) mice with a FVB background [36]. *Prnp*^*0/0*^ and Tg(MoPrP)/*Prnp*^*0/0*^ littermates used in this study were produced by intercross between the resulting Tg(MoPrP)/*Prnp*^*0/0*^ mice and the backcrossed *Prnp*^*0/0*^ mice. Tg(PrPΔOR)*/Prnp*^*0/0*^ mice with the C57BL/6 background were produced elsewhere [37]. Tg(PrPΔOR)*/Prnp*^*0/0*^ mice were maintained by intercrossing the backcrossed *Prnp*^*0/0*^ mice and Tg(PrPΔOR)*/Prnp*^*0/0*^ mice and used in this study. The Tg allele was detected by PCR using a primer pairs (SH3UT-S, 5’-tcggacgacaagagacaatc-3’; SHPA-A, 5’-taggggccacacagaaaaca-3’), which specifically amplifies the 3’ UTR region of the hamster PrP gene used in the Tg construct. The knockout allele was detected by PCR for the neomycin resistant gene using primer pairs (Neo163S, 5’-ggtgccctgaatgaactgca-3’; Neo390A, 5’-ggtagccggatcaagcgtat-3’). The PrP allele was detected by PCR using primer pairs (PrP-23aa-S, 5’-aaaaagcggccaaagcctgga-3’; PrP-231aa-AS, 5’-gctggatcttctcccgtcgtaataggcctg-3’).

### Virus preparation

IAV A/PR/8/34 (H1N1), A/Aichi/2/68 (H3N2), and A/WSN/33 (H1N1) were injected into the allantoic sac of 11-day-old chicken embryos in eggs and incubated at 36°C for 48 h. The eggs were chilled at 4°C for at least for 4 h prior to harvesting the allantoic fluids. Cellular debris in the allantoic fluids was removed by centrifugation at 2,380×g at 4°C for 30 min. The clarified allantoic fluids were layered over a 20% sucrose cushion and centrifuged at 25,000×g at 4°C for 120 min. The pellet containing viruses was suspended in phosphate-buffered saline (PBS), and stored in multiple aliquots at -80°C until used.

### Intranasal infection with IAVs

Male and female mice aged 5 weeks were intranasally inoculated with IAVs in a total volume of 20 μL (10 μL in each nasal cavity), and monitored for survival and weight loss for 14 days. The IAV stock aliquot was thawed and diluted in saline before used.

### Intranasal administration with LPS

Sixty micrograms of LPS (026:B6, Sigma-Aldrich, St Louis, MO) in 20 μL PBS were intranasally administered into a mouse using a micropipette (10 μL in each nasal cavity). PBS was similarly administered as a control.

### Treatment with BHA and allopurinol

BHA and allopurinol were purchased from Sigma-Aldrich. BHA was dissolved in dimethyl sulfoxide (DMSO) and stored at -20°C until use. BHA was orally administered at 200 mg/kg/day using a sonde needle from 2 to 5 dpi. According to the Material Safety Data Sheet (MSDS) (ScienceLab. com, Inc. Dickinson, Texas), the oral LD50 of BHA is 1,100 mg/kg in mice. Allopurinol was prepared in PBS and then stored at -20°C until use. Allopurinol was orally administered at 2 mg/kg/day using a sonde needle from -1 to 14 dpi. According to the MSDS (Sigma-Aldrich), the oral LD50 of allopurinol is 78 mg/kg in mice.

### Tissue and cell homogenization

Tissues were homogenized using a Polytron homogenizer (PT 2100, Brinkman Instruments, Inc., Westbury, NY) in 2 mL of PBS for measurement of cytokines and Cu ions, and in a lysis buffer (0.5% Triton X-100, 0.5% sodium deoxycholate, 150 mM NaCl, 50 mM Tris-HCl, pH 7.4, 1 mM EDTA) containing protease inhibitor cocktail (Nakalai Tesque, Kyoto, Japan) for Western blotting and for measurement of ROS levels and SOD activity. Cells were homogenized in a protease inhibitor cocktail (Nakalai Tesque)-containing lysis buffer and subjected to Western blotting and measurement of ROS levels and SOD activity. The homogenates were clarified by centrifugation at 1,000×g for 2 min at 4°C. Protein concentration of the homogenates was measured using the BCA method (Thermo Scientific, Rockford, IL).

### Determination of virus titers

Virus titers were expressed as IFU/mL. IFU/mL was determined using Madin-Darby canine kidney (MDCK) cells as follows. MDCK monolayer cells were incubated with 10-fold serial dilutions of each sample of interest for 14 h at 37°C. The cells were then fixed with 4% paraformaldehyde, permeabilized with 0.3% Triton-X100 in PBS, and immunostained with anti-NP monoclonal antibodies (GeneTex, Irvine, CA). Signals were visualized using horseradish peroxidase (HRP)-conjugated anti-rabbit IgG antibodies (GE Healthcare, Waukesha, WI) and True Blue Peroxidase Substrate (KPL, Gaithersburg, MD). IFU/mL was defined as the number of the cells positive for the anti-NP signals in 1 mL of each sample.

### Hematoxylin-eosin (H-E) staining

After euthanasia of mice, lungs were quickly removed, fixed with 4% paraformaldehyde, dehydrated, embedded in paraffin, and sliced into 5 μm-thick tissue sections. The sections were deparaffinized, rehydrated, and stained with hematoxylin for 5 min and eosin for 30 sec.

### Determination of atelectatic lung areas

The atelectatic lung area was evaluated using Photoshop software (Adobe, San Jose, CA) and ImageJ software (NIH, Bethesda, MD). Briefly, the original RGB color images of H-E stained lung sections were converted to black-on-white images using Photoshop software and saved in TIFF format. The binary images in TIFF format were again converted into a white-on-black image using the ImageJ application. Atelectatic lung area was expressed as the area of white pixels, which represent solid areas, against total lung area (white and black pixels).

### Immunofluorescence staining

Five micrometer-thick tissue sections on glass slides (Matsunami, Tokyo, Japan) coated with poly-L-lysin (Wako Pure Chemicals, Osaka, Japan) were deparaffinized and rehydrated, and treated with proteinase K (Wako Pure Chemicals, 20 mg/ml in 10 mM Tris HCl, pH 7.6) at 37°C for 30 min. After washed with PBS, the sections were incubated with primary antibodies against PrP (IBL-N, Immuno Biological Laboratories, Gunma, Japan), podoplanin (MBL, Nagoya, Japan), SP-C (Santa Cruz Biotechnology), CC10 (Santa Cruz Biotechnology), and NP virus protein (GeneTex) overnight at 4°C, and stained with Alexa Fluor 594 goat anti-rabbit IgG (Invitrogen) for IBL-N anti-PrP antibodies, Texas Red-X goat anti-rat IgG (Invitrogen) for anti-podoplanin antibody, Alexa Fluor 488 donkey anti-goat IgG (Invitrogen) for anti-SP-C and anti-CC10 antibody, and Alexa Fluor 488 goat anti-rabbit IgG (Invitrogen) for anti-NP antibody for 2 h at room temperature. The sections were mounted with CC/Mount (Diagnostic BioSystems, Pleasanton, CA) containing DAPI (Dojindo Laboratories, Kumamoto, Japan). Fluorescent images were visualized using BIOREVO BZ-9000 (Keyence, Osaka, Japan).

### TUNEL staining

TUNEL staining was performed using the *in situ* cell death detection kit and fluorescein (Roche Diagnostics, Mannheim, Germany) in accordance with the manufacturer’s protocol. In brief, the deparaffinized tissue sections were treated with 20 μg/mL proteinase K in 10 mM Tris-HCl for 30 min at room temperature and incubated in the TUNEL reaction mixture for 1 h at 37°C in a humidified dark chamber. The sections were washed with PBS for 5 min 3 times and signals were then detected using BIOREVO BZ-9000 (Keyence).

### Western blotting

Proteins in each sample were denatured by boiling for 5 min in Laemmli’s sample buffer and subjected to sodium dodecyl sulfate-polyacrylamide gel electrophoresis. Proteins were electrically transferred onto Immobilon-PVDF membranes (Millipore, Bedford, MA), and membranes were blocked for 2 h with 5% non-fat dry milk-containing TBST (0.1% Tween-20, 100 mM NaCl, 10 mM Tris-HCl, ph 7.6). Primary antibodies against PrP (6D11, COVANCE, Dedham, MA; SAF32, Cayman Chemical Company, Ann Arbor, MI; 3F4, BioLegend, San Diego, CA), pro-caspase 3 (Cell Signaling, Beverly, MA), the cleaved caspase 3 (Cell Signaling), NP (GeneTex), NS1 (GeneTex), HA (GeneTex), M2 (GeneTex), podoplanin (MBL), SP-C (Santa Cruz Biotechnology), CC10 (Santa Cruz Biotechnology), XO (Santa Cruz Biotechnology), SOD1 (Abcam, Cambridge, UK), SOD2 (Abcam) and β-actin (Sigma-Aldrich) were incubated with the membrane overnight at 4°C. Signals were visualized using HRP-conjugated anti-mouse IgG antibodies (GE Healthcare), anti-rabbit IgG antibodies (GE Healthcare), anti-rat IgG antibodies (GE Healthcare), or anti-goat IgG antibodies (R&D systems, Minneapolis, MN), and detected using a chemiluminescence image analyzer LAS-4000 mini (Fujifilm Co., Tokyo, Japan). Signal intensities were measured using ImageJ 64.

### Enzyme-linked immunosorbent assay (ELISA) for cytokines

IL-6, TNF-α, and IFN-γ levels in samples were determined using a Quantikine ELISA kit (R&D systems) according to the respective protocols provided by the manufacturer. In brief, the samples were diluted 1:1 with the assay diluent provided in the kit and added to the ELISA microplate wells. The protein standards for IL-6, TNF-α, and IFN-γ were also added to other wells. The plates were then left for 2 h at room temperature, and the wells were washed with wash buffer 5 times and mouse IL-6, TNF-α, or IFN-γ conjugate added followed by incubation for 2 h. The wells were then washed with the wash buffer and the substrate reagent added followed by incubation for 30 min. The reaction was stopped by addition of the stop solution. The optical density of each well was measured at 450 nm in an automated microplate reader (Thermo LabSystems, MA, USA). The amounts of IL-6, TNF-α, or IFN-γ in each sample were determined using the standard curve for the amounts of IL-6, TNF-α, or IFN-γ.

### ROS measurement

ROS concentration in samples was measured using an OxiSelect Intracellular ROS Assay Kit (Cell Biolabs, San Diego, CA). The assay uses 2’,7’-dichlorodihydrofluorescin diacetate (DCFH-DA), which is deacetylated to non-fluorescent 2’,7’-dichlorodihydrofluorescin and then oxidized by ROS to highly fluorescent 2’,7’-dichlorofluorescin (DCF). Each of the samples were mixed with 1×DCFH-DA solution in a 96-well black plate and incubated at 37°C for 48 h. ROS concentration in the samples was measured by determining the fluorescence intensities of DCF at 480 nm using Spectra Max Gemini EM (Molecular devices, Sunnyvale, CA).

### Measurement of SOD activity

SOD activity in samples was determined using an OxiSelect Superoxide dismutase activity assay kit (Cell Biolabs). This assay uses a xanthine/XO system to produce superoxide anions, which reduce chromagen to produce a formazan dye, which is colorimetrically detectable at 490 nm. SOD activity in the samples was determined as the inhibition of formazan dye production. Each of the samples was mixed with 1× XO solution in a 96-well black plate and incubated at 37°C for 60 min and the formazan dye produced was colorimetrically detected at 490 nm using Spectra Max Plus (Molecular devices). SOD1 inhibition was achieved by the addition of DDC (Sigma-Aldrich) to a final concentration of 1 mM into the mixture as described elsewhere [59–61].

### Measurement of Cu ions

Total copper levels in samples were assessed using the Metallo assay low copper LS kit (Metallogenics, Chiba, Japan) according to the manufacturer’s instructions. The pH of the samples was adjusted to 3.0 by adding a small amount of 0.25 mM HCl. Color reagent was then added and incubated at room temperature for 10 min. The copper concentration in the samples was calculated by measuring the absorbance at 580 nm using Spectra Max Plus (Molecular devices).

### Preparation of primary lung cell culture and viral infection

After euthanasia of mice, whole lungs were removed after perfusion of the mice with saline. The lungs were then cut into pieces and sieved through a 40 μm nylon cell strainer (BD Falcon, Franklin Lakes, NJ) with PBS. Lung cells were then collected by centrifugation at 1,000×g at 4°C for 2 min. The collected cells were suspended in Ham’s F-12K medium (Life Technologies, Grand Island, NY) supplemented with 15% FBS and cultured for 24 h. The cells were then cultured in F-12K medium without FBS in a 96-well plate at a density of 5.0×10^4^ cells/well for another 24 h, and infected with IAV/PR8 at a 1 MOI in the presence of 0.05% trypsin (Invitrogen). Cell viability was assessed using a Cell Counting Kit-8 (Dojindo).

### Knockdown of PrP^C^ expression and IAV infection in A549 cells

Human lung epithelial A549 cells were maintained in Dulbecco’s modified Eagle’s medium (DMEM, Wako Pure Chemicals) with 10% FBS and transfected with non-targeting control siRNA (cat: D-001210-01-05, Thermo Scientific) and human PrP-specific siRNA (cat: D-011101-02, Thermo Scientific). Briefly, 6.25 μL of RNAiMAX transfection reagent (Invitrogen) was mixed with 125 μL of Opti-MEM (Life Technologies) and incubated for 5 min at room temperature. In a separate tube, siRNA was added to 125 μL of Opti-MEM at a final concentration of 150 nM and the solution was then mixed with the RNAiMAX mixture for 20 min at room temperature. The siRNA/RNAiMAX mixture was then added to A549 cells in a 6-well plate. At 24 h after transfection, cells were washed with PBS and infected with IAV/PR8 at 1 MOI in 10% FBS-containing DMEM. Cells were collected, lysed, and subjected to Western blot analysis 24 h after infection.

### Preparation of splenocytes

After euthanasia of mice, whole spleens were removed and sieved through a 40 μm nylon cell strainer (BD Falcon) with PBS. Splenocytes were then harvested by centrifugation at 1,000×g for 2 min at 4°C. The resulting pellet was suspended in ACK buffer (0.15 M NH_4_Cl, 1.0 mM KHCO_3_, 0.1 mM Na_2_EDTA, pH 7.2) at room temperature for 2 min to disrupt red blood cells and centrifuged at 1,000×g for 2 min at 4°C. The collected splenocytes were adjusted to a concentration of 2.5×10^6^ cells/mL in RPMI 1640 medium (Sigma-Aldrich) supplemented with 10% FBS, 1% L-glutamine (Sigma-Aldrich), 2 μM L-glutamate (Sigma-Aldrich), non-essential amino acids (Sigma-Aldrich), 10 mM HEPES, and 1 mM sodium pyruvate.

### RT-PCR

RT-PCR was performed using OneStep RT-PCR Kit (QIAGEN, Hilden, Germany) according to manufacturer’s recommendations. Total RNA was first extracted from tissues using RNeasy Mini Kit (QIAGEN). Tissue homogenates in buffer RLT were transferred to a QIAshredder spin column (QIAGEN). The flow-through was mixed with 1 volume of 70% ethanol and then transfer to an RNeasy spin column (QIAGEN). Total RNA bound to the membrane was washed with buffer RW1 and then with buffer RPE, and eluted with RNase-free water. Eight ng of total RNA was then mixed with primers, dNTPs and OneStep RT-PCR enzyme mix. The mixture was incubated at 50°C for 30 min at RT and then subjected to PCR reaction (Initial PCR activation step at 95°C for 15 min; 3-step cycling: Denaturation at 94°C for 30 sec, Annealing at 56°C for 30 sec, Extension at 72°C for 1 min; Final extension at 72°C for 10 min). Sequences of the primers used and the number of PCR cycles used for each gene examined are given in S1 Table. The products were analyzed by 2% agarose gel electrophoresis.

### Determination of IAV/PR8-specific IgG and IgM titers

IAV/PR8-specific IgG and IgM titers in plasma were determined by ELISA. Each well of a 96 well immunoplate (Thermo Fisher Scientific, Roskilde, Denmark) were coated with the already prepared split IAV/PR8 vaccine [62] in PBS overnight at 4°C. The wells were then washed with PBS 3 times and blocked with PBS containing 4% Block Ace (Megmilk Snow Brand Co., Ltd., Hokkaido, Japan) for 1 h at 37°C. Mouse plasma samples were first diluted to 1:16 and subsequently 1:2 in PBS and added to the wells at 37°C for 4 h. The wells were washed with PBS containing 0.05% Tween-20 3 times, and immune complexes were detected using HRP-conjugated goat anti-mouse IgM or IgG antibodies (Bethyl Laboratories, Inc., Montgomery, TX) and 1-Step Ultra TMB-ELISA (Thermo Scientific). The signals were detected spectrophotometrically at 450 nm using Spectra Max Plus (Molecular devices). Antibody titers are defined as the reciprocal of the highest dilution of samples for which the optical density was at least twice of that of the negative control samples, and are expressed as reciprocal log_2_ titers.

### ELISPOT assay

ELISPOT assay for TNF-α- and IFN-γ-secreting cells in lungs and spleens was performed using Mouse TNF-α ELISpot^BASIC^ (HRP) kit (Mabtech Inc., Cincinnati, OH) and Mouse IFN-γ ELISpot^BASIC^ (HRP) kit (Mabtech Inc.), respectively, according to the manufacturer’s recommendations. In brief, each well of the 96-well nitrocellulosebottomed Millititer HA plate (Millipore) was coated with capture MT1C8/23C9 anti-TNF-α antibody (Mabtech Inc.) and capture AN18 anti-IFN-γ antibody (Mabtech Inc.) in PBS overnight at 4°C. Primary lung cells (4×10^4^ cells/well) and splenocytes (2.5×10^5^ cells/well) from mice at 14 dpi were added to the wells and stimulated with 5 nM of the IAV peptide PA224-233 (Anaspec Inc., Fremont, CA) for 3 days at 37°C in a humidified chamber with 5% CO_2_ in air. The wells were then washed with PBS 5 times and immune complexes were detected with biotin-conjugated detection antibodies (Mabtech Inc., MT11B10-biotin for TNF-α and R4-6A2-biotin for IFN-γ), Streptavidin-HRP (Mabtech Inc.), and 1-Step Ultra TMB-ELISA (Thermo Scientific). The number of the spots in the wells was quantified using a computerized ELISPOT reader (CTL-Immunospot S6 analyzer, Cellular Technology Ltd., Cleveland, OH).

### Statistical analysis

Survival rates were analyzed using the log-rank test. All other data were analyzed using the Student’s *t*-test.

## Supporting information

S1 Table List of genes, sequences of the primers, and the number of cycles used for RT-PCR gene expression analysis. (DOCX)

S1 Fig Similar expression levels of PrP^C^ in the lungs of male and female WT mice. Left panel: Western blotting of the lungs of male and female WT mice with 6D11 anti-PrP antibody. Right panel: Quantification of the intensity for PrP^C^ after normalization against that for β-actin (Actb). Signal intensity of PrP^C^ in female lungs was evaluated against that in male lungs. NS, not significant. Error bars, standard deviation (SD).
(TIF)

S2 Fig Active innate and adaptive immune responses against IAV/PR8 infection in *Prnp^0/0^* mice. (A) Upper panels: RT-PCR gene expression analysis on agarose gels of innate immunity-related genes and the viral NP gene in the lungs of *Prnp*^*0/0*^ and WT mice uninfected (Un) and infected with IAV/PR8 (50 IFU) at 3 and 5 dpi. Lower panels: Quantification of the signal intensity for each of the genes against that of uninfected (Un) control WT. (B) Serum levels of IAV/PR8-specific IgM and IgG in *Prnp*^*0/0*^ (n = 3) and WT (n = 3) mice administrated with saline as uninfected controls or with IAV/PR8 (50 IFU) at 10 and 14 dpi. (C) ELISPOT analysis for TNF-α- or IFN-γ-secreting cells in the lungs and spleens of *Prnp*^*0/0*^ (n = 3) and WT (n = 3) mice administrated with saline as uninfected controls or with IAV/PR8 (50 IFU) at 14 dpi. *, p<0.05; **, p<0.01. NS, not significant. Error bars, SD.
(TIF)

S3 Fig Higher vulnerability of *Prnp^0/0^* primary lung cells to IAV/PR8 infection. (A) Triplicate analysis for cell viability of WT, *Prnp*^*0/0*^, and Tg(MoPrP)/*Prnp*^*0/0*^ primary lung cells 2 days after infection with (+) or without (-) IAV/PR8 at 1 MOI. (B) Western blotting of WT, *Prnp*^*0/0*^, and Tg(MoPrP)/*Prnp*^*0/0*^ primary lung cells 2 days after infection with (+) or without (-) IAV/PR8 at 1 MOI for the viral proteins NP, HA and M2, pro-caspase 3, and the cleaved caspase 3 fragments. Actb is an internal control. (C) Western blotting of A549 cells treated with control (Ctr) and human PrP-specific siRNAs 24 h after infection with (+) or without (-) IAV/PR8 at 1 MOI for PrP^C^, the viral proteins NP, HA and M2, pro-caspase 3, and the cleaved caspase 3 fragments. Actb is an internal control. (D) Triplicate analysis for DCF levels in WT, *Prnp*^*0/0*^, and Tg(MoPrP)/*Prnp*^*0/0*^ primary lung cells 2 days after infection with (+) or without (-) IAV/PR8 at 1 MOI. (E) Triplicate analysis for SOD activity in WT, *Prnp*^*0/0*^, and Tg(MoPrP)/*Prnp*^*0/0*^ primary lung cells 2 days after infection with (+) or without (-) IAV/PR8 at 1 MOI. *, p<0.05; **, p<0.01. Error bars, SD.
(TIF)

S4 Fig Similar lung injuries in WT and *Prnp^0/0^* mice after intranasal administration with LPS. (A) Mortality and body weight of *Prnp*^*0/0*^ and WT mice after intranasal administration with LPS (*Prnp*^*0/0*^, n = 5; WT, n = 5). (B) Left panels: Western blotting of the lungs of WT and *Prnp*^*0/0*^ mice 24 h after administration with (+) or without (-) LPS for pro-caspase 3 and its cleaved fragments. Actb is an internal control. Right panel: Quantification of the signal intensity for the cleaved caspase 3 fragments against that for LPS-untreated WT. (C) TNF-α and IFN-γ levels in the lungs of WT (n = 3) and *Prnp*^*0/0*^ (n = 3) mice 24 h after administration with (+) or without (-) LPS. NS, not significant. Error bars, SD.
(TIF)
