# Supplementary material for: Prion protein protects mice from lethal infection with influenza A viruses
Source: PLoS Pathog. 2018 May 3;14(5):e1007049. doi: 10.1371/journal.ppat.1007049 (PMC5953499; doi:10.1371/journal.ppat.1007049)
Supplement: S1 Table — (DOCX) [file ppat.1007049.s001.docx]

**S1 Table. List of genes, sequences of the primers, and the number of cycles used for RT-PCR gene expression analysis**

| **Gene** | **Primer** | **Sequence** | **Cycles** |
| --- | --- | --- | --- |
| RIG-I | sense  antisense | 5’-GGA GAA GAT GGC CGA ATG TC-3’  5’-GCG AAG AAG ACC ACT TTC CC-3’ | 30 |
| MDA5 | sense  antisense | 5’-GGC ACC ATG GGA AGT GAT TC-3’  5’-ACC GTC ATC GTC TCC ACT CT-3’ | 25 |
| TNF-α | sense  antisense | 5’-GCC TAT GTC TCA GCC TCT TC-3’  5’-GGA GGT TGA CTT TCT CCT GG-3’ | 20 |
| IFN-α | sense  antisense | 5’-TGG CTA GGC TCT GTG CTT TC-3’  5’-AGCTGC TGG TGG AGG TCA TT-3’ | 25 |
| IFN-γ | sense  antisense | 5’-CTT GGC TTT GCA GCT CTT CC-3’  5’-GCT CAT TGA ATG CTT GGC GC-3’ | 30 |
| NP | sense  antisense | 5’-GTT GGA ACA ATG GTG ATG GA-3’  5’-TGA TTA GGC TGT ACA CTT GGC-3’ | 15 |
| Actb | sense  antisense | 5’-GGG AGA GCA TAG CCC TCG TAG AT-3’  5’-CCT TCA ACA CCC CAG CCA TGT AC-3’ | 20 |
