# Supplementary figures and images for: Prion protein protects mice from lethal infection with influenza A viruses
Source: PLoS Pathog. 2018 May 3;14(5):e1007049. doi: 10.1371/journal.ppat.1007049 (PMC5953499; doi:10.1371/journal.ppat.1007049)

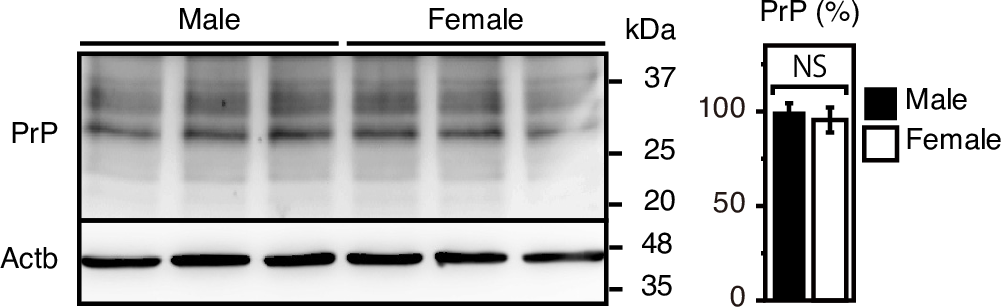

Supplement: S1 Fig — Left panel: Western blotting of the lungs of male and female WT mice with 6D11 anti-PrP antibody. Right panel: Quantification of the intensity for PrPC after normalization against that for β-actin (Actb). Signal intensity of PrPC in female lungs was evaluated against that in male lungs. NS, not significant. Error bars, standard deviation (SD). (TIF) [file ppat.1007049.s002.tif]

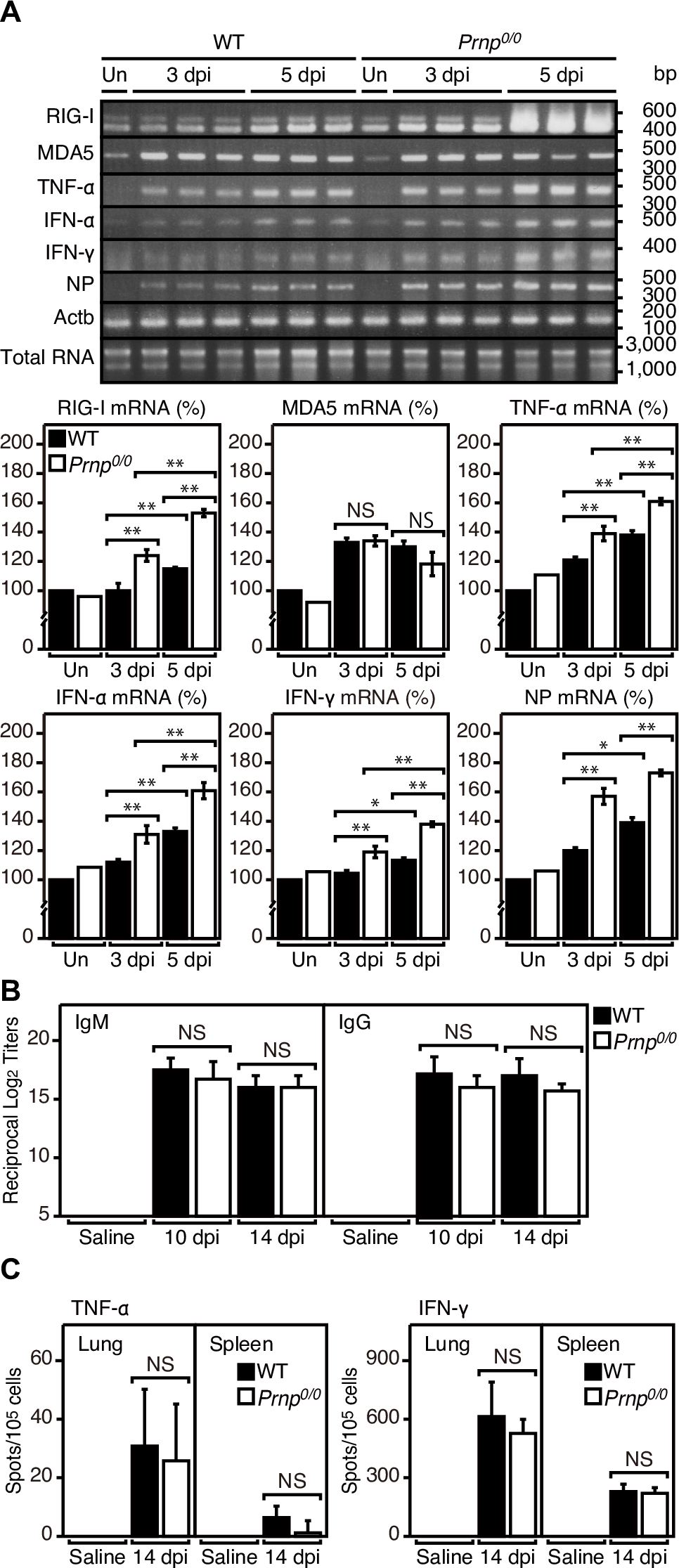

Supplement: S2 Fig — (A) Upper panels: RT-PCR gene expression analysis on agarose gels of innate immunity-related genes and the viral NP gene in the lungs of Prnp0/0 and WT mice uninfected (Un) and infected with IAV/PR8 (50 IFU) at 3 and 5 dpi. Lower panels: Quantification of the signal intensity for each of the genes against that of uninfected (Un) control WT. (B) Serum levels of IAV/PR8-specific IgM and IgG in Prnp0/0 (n = 3) and WT (n = 3) mice administrated with saline as uninfected controls or with IAV/PR8 (50 IFU) at 10 and 14 dpi. (C) ELISPOT analysis for TNF-α- or IFN-γ-secreting cells in the lungs and spleens of Prnp0/0 (n = 3) and WT (n = 3) mice administrated with saline as uninfected controls or with IAV/PR8 (50 IFU) at 14 dpi. *, p<0.05; **, p<0.01. NS, not significant. Error bars, SD. (TIF) [file ppat.1007049.s003.tif]

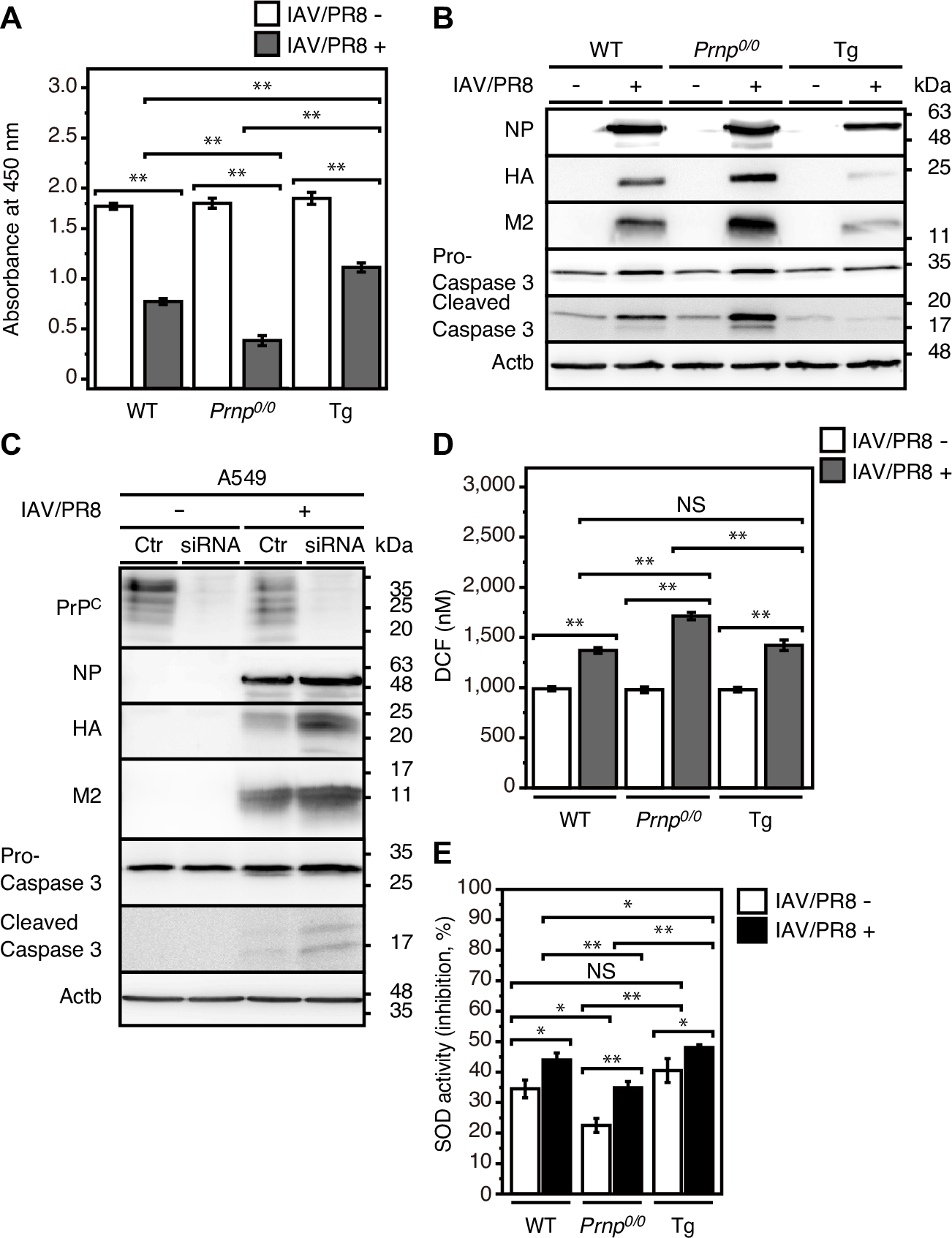

Supplement: S3 Fig — (A) Triplicate analysis for cell viability of WT, Prnp0/0, and Tg(MoPrP)/Prnp0/0 primary lung cells 2 days after infection with (+) or without (-) IAV/PR8 at 1 MOI. (B) Western blotting of WT, Prnp0/0, and Tg(MoPrP)/Prnp0/0 primary lung cells 2 days after infection with (+) or without (-) IAV/PR8 at 1 MOI for the viral proteins NP, HA and M2, pro-caspase 3, and the cleaved caspase 3 fragments. Actb is an internal control. (C) Western blotting of A549 cells treated with control (Ctr) and human PrP-specific siRNAs 24 h after infection with (+) or without (-) IAV/PR8 at 1 MOI for PrPC, the viral proteins NP, HA and M2, pro-caspase 3, and the cleaved caspase 3 fragments. Actb is an internal control. (D) Triplicate analysis for DCF levels in WT, Prnp0/0, and Tg(MoPrP)/Prnp0/0 primary lung cells 2 days after infection with (+) or without (-) IAV/PR8 at 1 MOI. (E) Triplicate analysis for SOD activity in WT, Prnp0/0, and Tg(MoPrP)/Prnp0/0 primary lung cells 2 days after infection with (+) or without (-) IAV/PR8 at 1 MOI. *, p<0.05; **, p<0.01. Error bars, SD. (TIF) [file ppat.1007049.s004.tif]

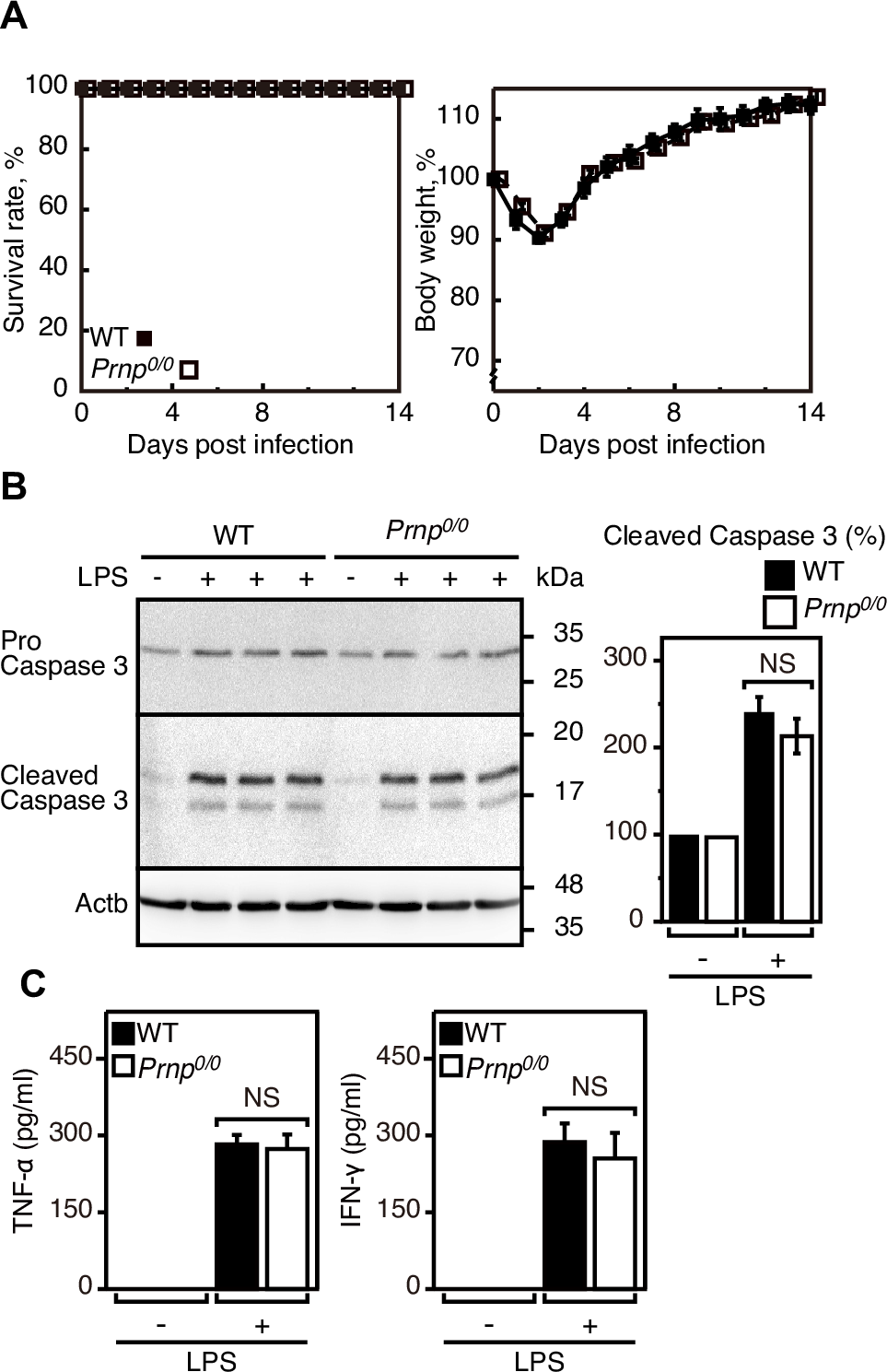

Supplement: S4 Fig — (A) Mortality and body weight of Prnp0/0 and WT mice after intranasal administration with LPS (Prnp0/0, n = 5; WT, n = 5). (B) Left panels: Western blotting of the lungs of WT and Prnp0/0 mice 24 h after administration with (+) or without (-) LPS for pro-caspase 3 and its cleaved fragments. Actb is an internal control. Right panel: Quantification of the signal intensity for the cleaved caspase 3 fragments against that for LPS-untreated WT. (C) TNF-α and IFN-γ levels in the lungs of WT (n = 3) and Prnp0/0 (n = 3) mice 24 h after administration with (+) or without (-) LPS. NS, not significant. Error bars, SD. (TIF) [file ppat.1007049.s005.tif]
